# Chemical Compositions and Experimental and Computational Modeling of the Anticancer Effects of Cnidocyte Venoms of Jellyfish *Cassiopea andromeda* and *Catostylus mosaicus* on Human Adenocarcinoma A549 Cells

**DOI:** 10.3390/md21030168

**Published:** 2023-03-07

**Authors:** Afshin Zare, Alireza Afshar, Arezoo Khoradmehr, Neda Baghban, Gholamhossein Mohebbi, Alireza Barmak, Adel Daneshi, Afshar Bargahi, Iraj Nabipour, Sahar Almasi-Turk, Alireza Arandian, Mohammad Ismail Zibaii, Hamid Latifi, Amin Tamadon

**Affiliations:** 1 Student Research Committee, Bushehr University of Medical Sciences, Bushehr 75, Iran; 2 PerciaVista R&D Co., Shiraz 73, Iran; 3 The Persian Gulf Marine Biotechnology Research Center, The Persian Gulf Biomedical Sciences Research Institute, Bushehr University of Medical Sciences, Bushehr 73, Iran; 4 Food Lab, Bushehr University of Medical Sciences, Bushehr 73, Iran; 5 Department of Anatomical Sciences, School of Medicine, Bushehr University of Medical Sciences, Bushehr 73, Iran; 6 Laser and Plasma Research Institute, Shahid Beheshti University, Tehran 11, Iran; 7 Department of Physics, Shahid Beheshti University, Tehran 11, Iran

**Keywords:** jellyfish, apoptosis, venom, pulmonary adenocarcinoma, molecular docking

## Abstract

Nowadays, major attention is being paid to curing different types of cancers and is focused on natural resources, including oceans and marine environments. Jellyfish are marine animals with the ability to utilize their venom in order to both feed and defend. Prior studies have displayed the anticancer capabilities of various jellyfish. Hence, we examined the anticancer features of the venom of *Cassiopea andromeda* and *Catostylus mosaicus* in an in vitro situation against the human pulmonary adenocarcinoma (A549) cancer cell line. The MTT assay demonstrated that both mentioned venoms have anti-tumoral ability in a dose-dependent manner. Western blot analysis proved that both venoms can increase some pro-apoptotic factors and reduce some anti-apoptotic molecules that lead to the inducing of apoptosis in A549 cells. GC/MS analysis demonstrated some compounds with biological effects, including anti-inflammatory, antioxidant and anti-cancer activities. Molecular docking and molecular dynamic showed the best position of each biologically active component on the different death receptors, which are involved in the process of apoptosis in A549 cells. Ultimately, this study has proven that both venoms of *C. andromeda* and *C. mosaicus* have the capability to suppress A549 cells in an in vitro condition and they might be utilized in order to design and develop brand new anticancer agents in the near future.

## 1. Introduction

Among all classes of cancers, lung cancers have a high rate of mortality [1]. The fatality rate of lung cancer is more than the summation of the fatality rate of colon, breast, and pancreatic cancers [2]. Despite all progresses in the treatment of lung cancer, almost 50 percent of patients who suffer from it die within the first year after diagnosis and its five years survival rate is about 15 percent [3]. Pulmonary adenocarcinoma is the most common form of lung cancer [4]. About 40 percent of all lung cancer patients suffer from pulmonary adenocarcinoma [2], which has a high mortality and has a poor prognosis condition [5]. Additionally, various treatment strategies like chemotherapy, radiotherapy and surgery, which are considered for pulmonary adenocarcinoma, have some serious weak points [2]. New strategies for curing pulmonary adenocarcinoma have been suggested [6]. One of these strategies is to use natural products such as marine-derived components from various marine creatures [7]. Marine creatures are known as producers of materials that have effective pharmacological applications like anticancer biological activity [8].

Cnidarians, which are known as the most prevalent venomous marine animals, are one of the marine sources of biological compounds with various biological activities. The venom of cnidarians provides this ability to attack either attackers or victims [9]. Numerous tentacles in cnidarians’ bodies have a specialized cell in their structure, called cnidocyte. Cnidocytes are packages of poison with various compounds and macromolecules. When cnidocytes undergo physical stimulation, they throw their string-like structure in order to inject their poisonous content into the attacker or prey and this mechanism is named envenomation [10]. In some cnidarians’ species, the venom of cnidarians creates serious concerns for human health and can cause medical urge conditions [11]. Their venom has a wide range of effects on the human body, from local irritation to systemic reaction. These venoms have been investigated in various studies for different purposes, including examination of anticancer impacts, neurotoxin features, hemolytic effects, antimicrobial activity, etc. [12].

Jellyfish belongs to the phylum Cnidaria [13]. They have umbrella shape bells, oral arms and tentacles in their structure. Tentacles are known as the place of storage of the venom of jellyfish [13]. The venom of jellyfish contains various components, including enzymes, peptides, hemolysin, cytolysin, and neurotoxin [14]. Various biological features of the venom of jellyfish have been investigated, such as anti-microbial, cytotoxic and antioxidant activities. While the anti-cancer function of the extraction of jellyfish has not been examined on a vast scale, recent researches have demonstrated the potential anticancer effect of the venom of a jellyfish *Stomolophus nomurai* on human erythroleukemic cells (K562) [13], the venom of *Nemopilema nomurai* on hepatocarcinoma cells (HepG2) [15], and the venom of *Acromitus flagellatus* on human pulmonary adenocarcinoma cells (A549) [16].

*Cassiopea andromeda* is a member of the family of jellyfish and belongs to the Scyphozoa subgroup. Its habitat is in tropical inshore marine waters with shallow depths. The presence of *C. andromeda* is usually associated with mangrove populations [17]. *C. andromeda* is called upside-down jellyfish because it locates it on its umbrella-shaped bells in its habitat with its arms facing the sky. The worldwide spreading of the genus of *C. andromeda* is located in the Western Atlantic and Indo-Pacific tropical areas. *C. andromeda* exists in the Persian Gulf [18]. *C. andromeda* uses its cnidocytes for hunting prey, and like other cnidarians, its cnidocytes are a kind of storage place for its venom. This venom provides the ability to attack prey and capture them. Some of the features of the venom of *C. andromeda* have been studied in recent years [9]. The venom of *C. andromeda* created a necrosis condition in the derma, increasing the permeability of the vessels and causing hemorrhages [19].

*Catostylus mosaicus (Rhizostomeae)* is a scyphozoan jellyfish that lives in bays and estuaries. *C. mosaicus* lives in the Persian Gulf [20]. They gather on the surface of the water in their habitats [21]. *C. mosaicus* has venom in its cnidocytes [22]. The venom of *C. mosaicus* mainly causes pain and minor skin irritations [22]. Itchy rash and allergic reactions may only occur in highly sensitive persons [22]. Furthermore, the venom of *C. mosaicus* has anticancer potential and can be used for anticancer purposes [23].

In the present study, the anatomical structure of distribution of cnidocytes and cnidosomes in the body of two species of jellyfish, *C. andromeda* and *C. mosaicus,* was investigated. Additionally, the two-dimensional and three-dimensional structures of their cnidocytes and cnidosomes were evaluated. After separating and purifying cnidocytes cells, their venom was isolated in order to evaluate the apoptotic effects of the venoms on A549 cells in in vitro condition. Finally, by computational modeling, the apoptotic molecules were detected.

## 2. Results

### 2.1. GC-MS Findings of Venoms of C. andromeda and C. mosaicus

**Figure 1:**
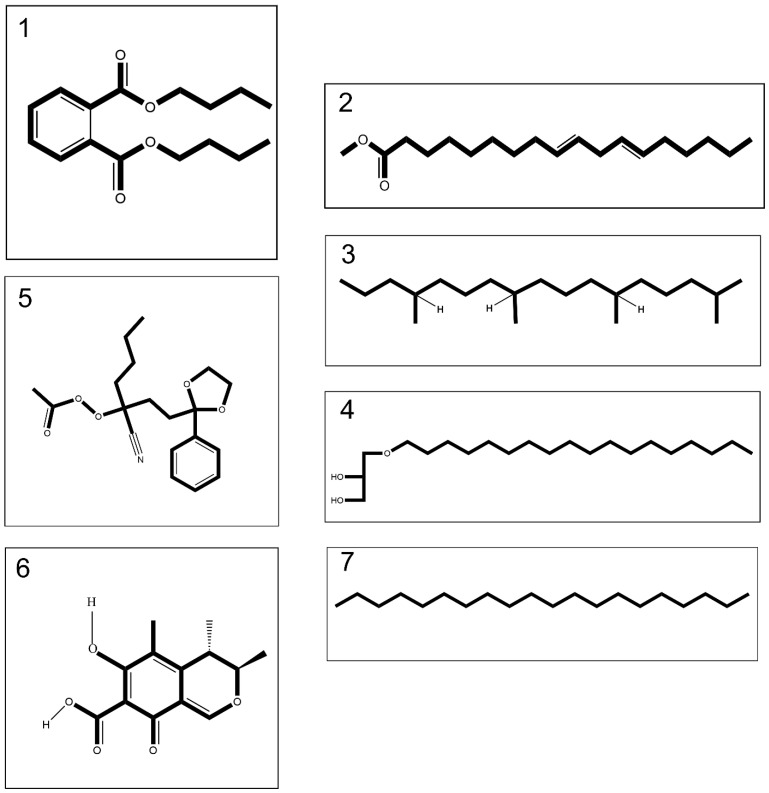
Compounds **1**–**4** merely tentatively identified from the venom of *Cassiopea andromeda* and *Catostylus mosaicus*, compound **5** merely tentatively identified from the venom of *C. andromeda* and compounds **6** and **7** merely tentatively identified from the venom of *C. mosaicus*, including dibutyl phthalate (**1**), 9,12-octadecadienoic acid, methyl ester (**2**), heptadecane,2,6,10,14-tetramethyl- (**3**), batilol (**4**), ethaneperoxoic acid, 1-cyano-1-[2-(2-phenyl-1,3-dioxolan-2-yl)ethyl]pentyl ester (**5**), citrinin (**6**) and eicosane (**7**).

The extraction process by means of methanol: Chloroform: n-hexane (2:2:1 *v*/*v*) of both lyophilized crude venoms led to isolation of 10% (*w*/*w*) organic extract relative to the whole venom. GC-MS analyses detected 18 compounds in the venom of *C. andromeda* (Figure S1A and Table S1). Among these compounds, five compounds have anti-cancer and apoptotic effects based on prior studies (Figure 1 and Table 1). In addition, 15 compounds were detected using GC-MS analysis in the venom of *C. mosaicus* (Figure S1B and Table S2). Among these compounds, six compounds have anti-cancer and apoptotic effects based on previous studies (Figure 1 and Table 1). The effects of both venoms of *C. andromeda* and *C. mosaicus* on some cell lines are also listed in Tables S1 and S2. Additionally, other biological activities were observed from the components, which was detected by GC-MS in both of the venoms of *C. andromeda* and *C. mosaicus,* including anti-oxidant, oxidative stress, apoptosis, anti-inflammatory, inflammation, antimicrobial, and anti-viral activities (Tables S1 and S2).

### 2.2. Docking Findings

Ten conformations resulted from the interaction of each ligand with the selected target in the Western blot assay (please refer to Section 2.5. Western blot assay findings) after molecular docking with Autodock Vina. Among them, those with the highest negative (lowest) binding affinity (kcal/mol) were chosen as the best ones. The binding affinities of selected conformations are listed in Table 2.

**Figure 2:**
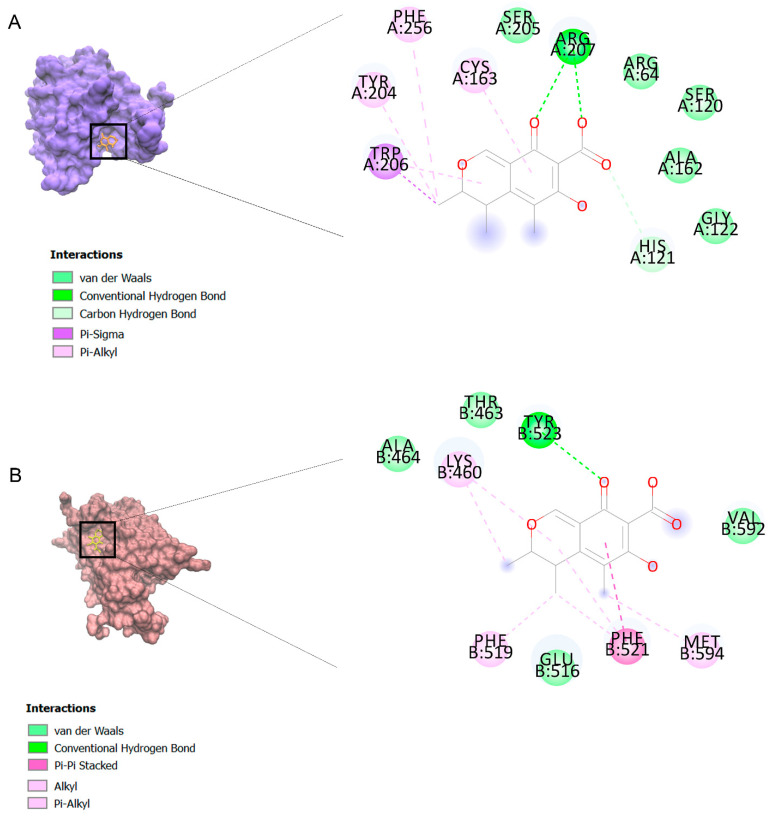

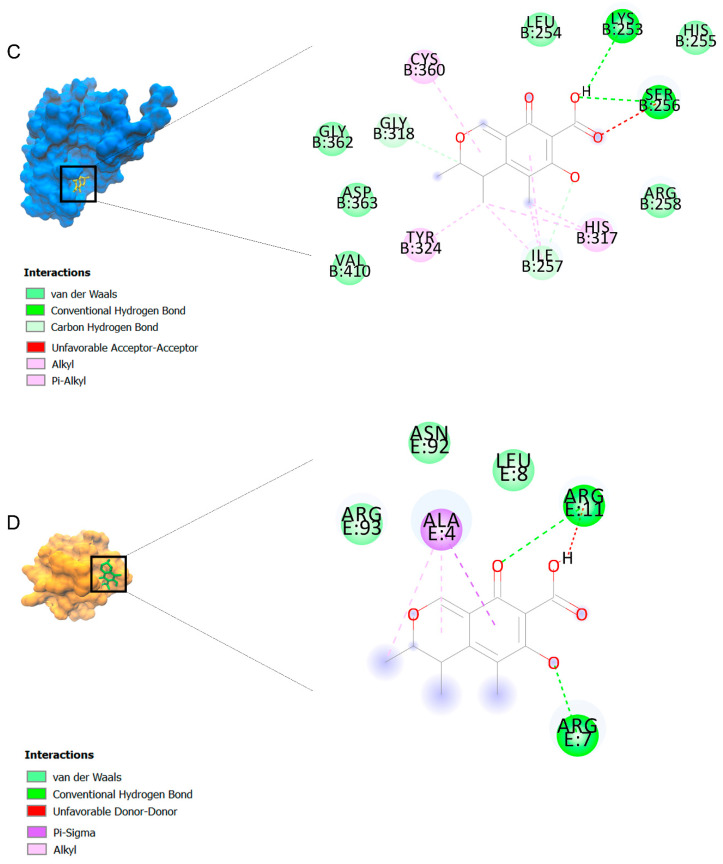

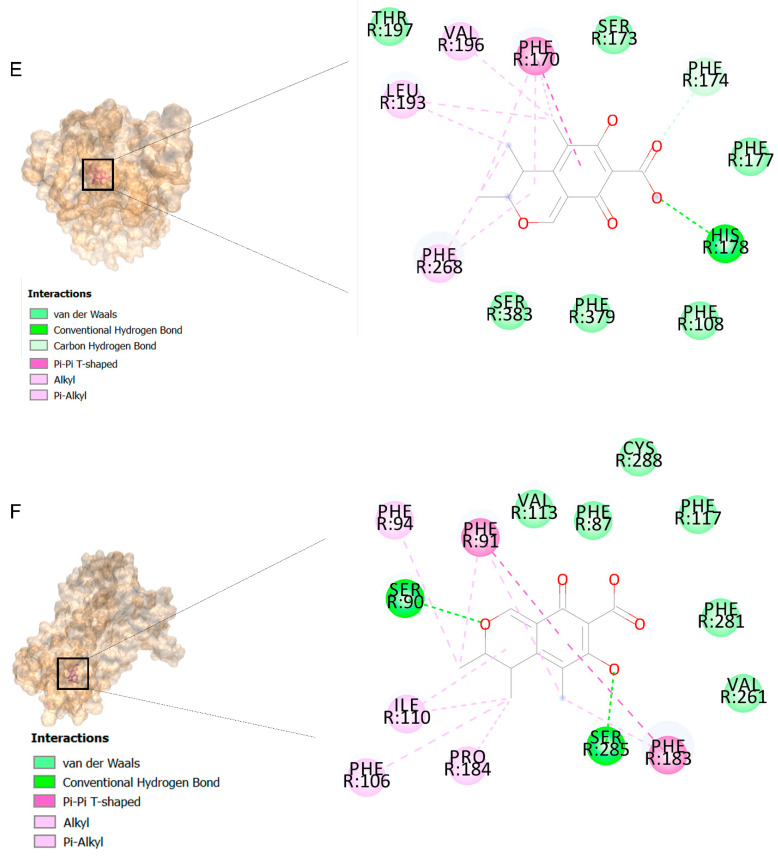

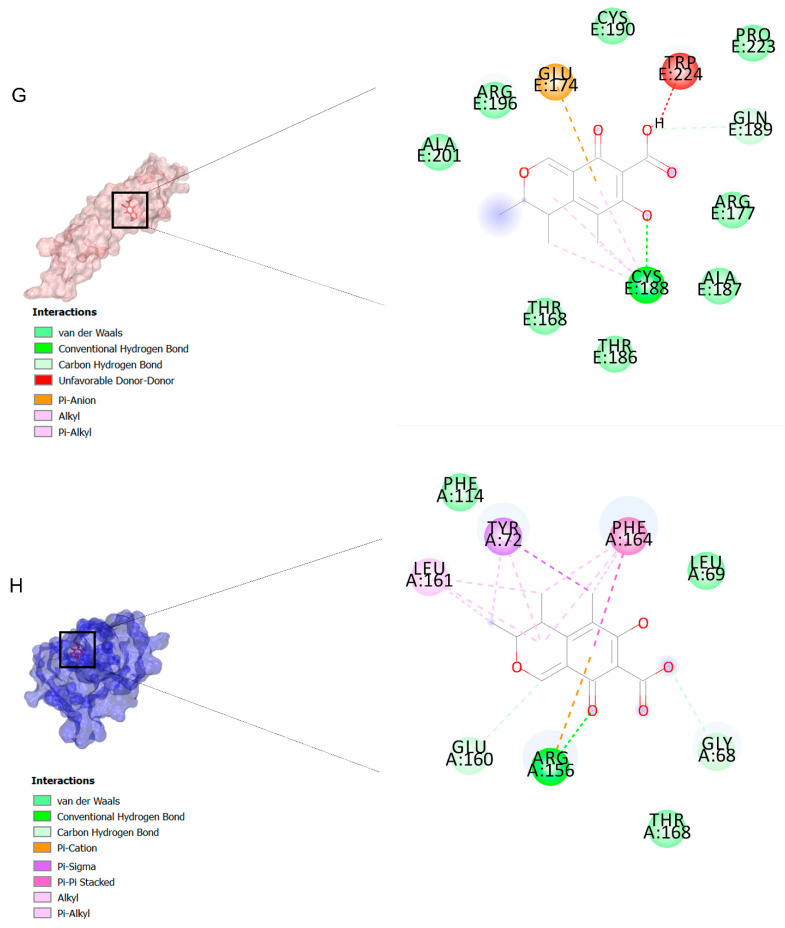

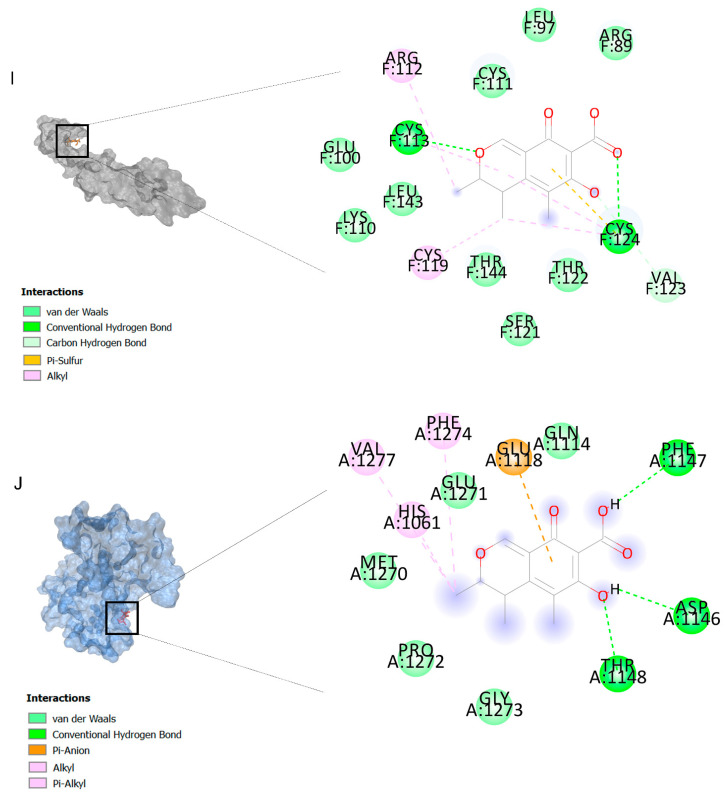

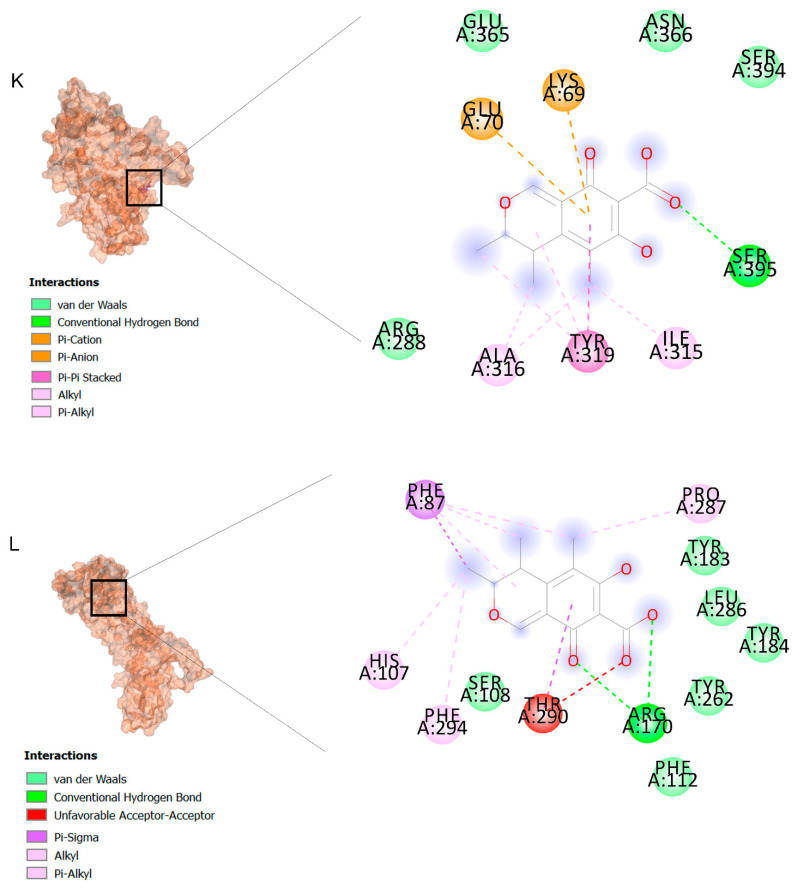

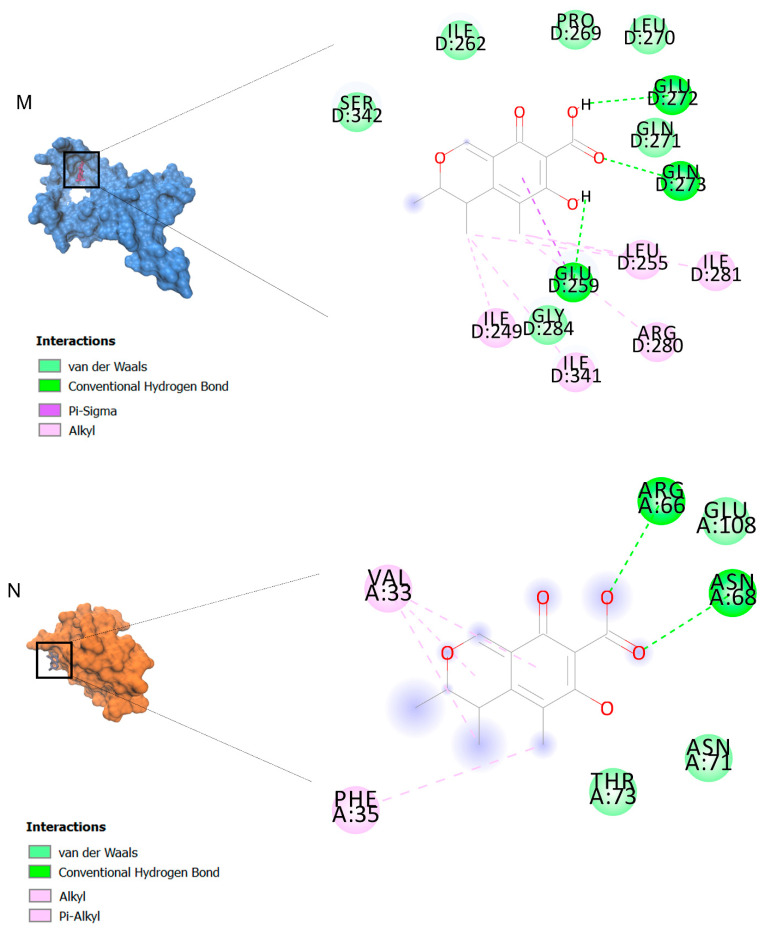

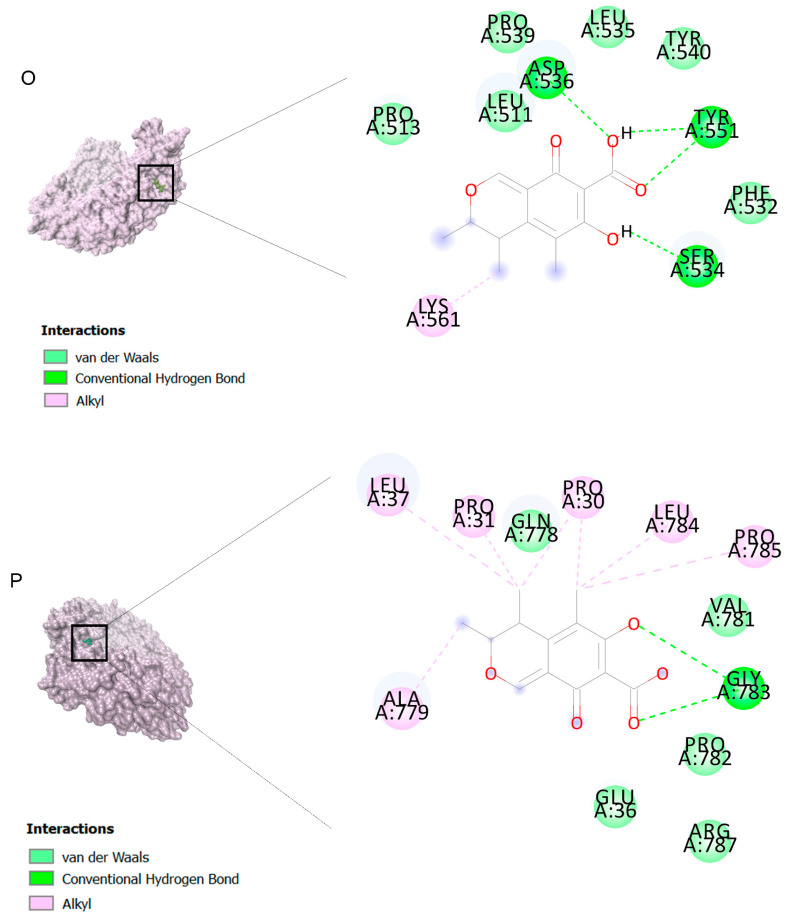

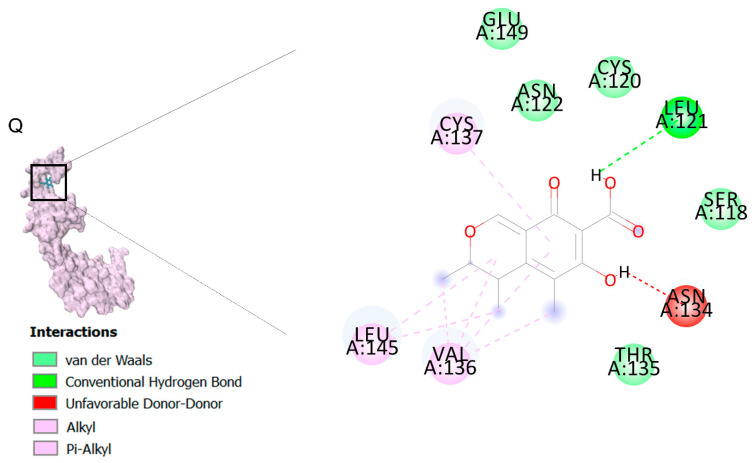
Computational modeling of the interaction of citrinin, a bioactive venom compound of *Cassiopea andromeda* and *Catostylus mosaicus* on apoptosis peptides in A549 demonstrating by the three-dimensional plot of the binding sites and the two-dimensional plot of interactions. Interactions of citrinin with (**A**) caspase-3, (**B**) caspase-7, (**C**) caspase-8, (**D**) caspase-9, (**E**) Cannabinoid receptor type 1 (CB1), (**F**) Cannabinoid receptor type 2 (CB2), (**G**) Death receptor 4 (DR4), (**H**) Endothelial protein C receptor (EPCR), (**I**) Fas receptor, (**J**) insulin-like growth factor 1 receptor (IGF1R), (**K**) metabotropic glutamate receptors (mGluRs), (**L**) prostaglandin D2 (PGD2) (**M**) Peroxisome proliferator-activated receptor gamma (PPAR-γ), (**N**) Transforming Growth Factor Beta Receptor 2 (TGFBR2), (**O**) Toll-like receptor 4 (TLR4), (**P**) Toll-like receptor 9 (TLR9), and (**Q**) Tumor necrosis factor receptor 1 (TNFR1).

The binding affinities are between −3 and −8.5 kcal/mole related to the interaction between eicosane with Death receptor 4 (DR4) and citrinin with Prostaglandin D2 (PGD2). Citrinin and ethaneperoxoic acid, 1-cyano-1-[2-(2-phenyl-1,3-dioxolan-2-yl)ethyl]pentyl ester showed better affinity to the targets. Figure 2 shows the intermolecular interactions of citrinin with the targets.

### 2.3. Molecular Dynamic (MD) Findings

The citrinin, which showed the highest affinity to the targets, was subjected to molecular dynamics. 3D structures of complexes of citrinin with targets obtained through molecular dynamics (MD) are shown in Figure 2. Additionally, RMSDs listed in Table 3 demonstrate the stability of citrinin complexes during 10 runs of molecular dynamic simulation. A lower RMSD value indicates greater stability of the protein complex. As observed, the RMSDs are in the range of 0.432–0.821 Å. It can be seen that the complex of citrinin with PGD2 with a lower RMSD value is more stable compared to other ones. This finding is in accordance with those predicted through the docking simulations.

### 2.4. MTT Assay Showed Anti-Proliferative Effects of Venoms of C. andromeda and C. mosaicus on A549 Cells

From one Kg of arm tissue of *C. andromeda*, we could isolate 3760 µg of venom. In *C. mosaicus*, the same amount of tissue had 6000 µg of venom. The isolated venoms were lyophilized and stored at −80 °C.

**Figure 3:**
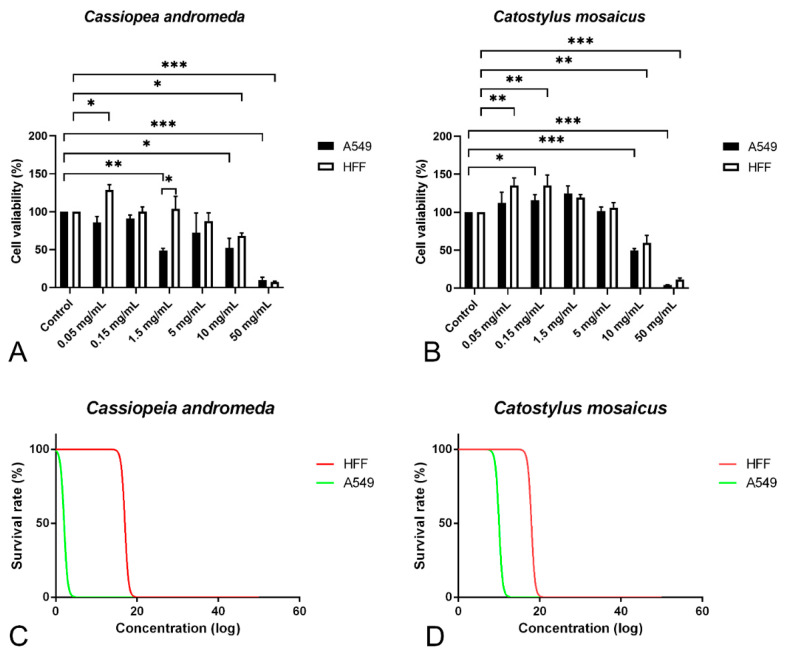
MTT proliferation assay and survival rate of A549 and human foreskin fibroblast (HFF) cell lines after exposure to different concentrations of the venom of *Cassiopea andromeda* and *Catostylus mosaicus*. MTT proliferation assay of A549 and HFF cell lines after exposure to *Cassiopea andromeda* (**A**) and *Catostylus mosaicus* (**B**). Lines above the columns showed differences between treatments and control (* *p* < 0.05, ** *p* < 0.01 and *** *p* < 0.001). Cytotoxic activity of the venom of *Cassiopea andromeda* (**C**) and *Catostylus mosaicus* (**D**) on A549 and HFF cell lines.

In order to study the anti-proliferative effect of the venom of *C. andromeda* and *C. mosaicus*, MTT assay was performed on A549 cell line and human foreskin fibroblast (HFF) cell line as control at concentrations of 0.05, 0.15, 1.5, 10 and 50 mg/mL. Venom of *C. andromeda* had anti-proliferative effect on A549 cell lines at the concentration of 1.5, 10 and 50 mg/mL (Figure 3A, *p* values are 0.008, 0.012 and 0.000, respectively). However, the venom of *C. mosaicus* had the anti-proliferative effect on A549 cell lines at 10 and 50 mg/mL (Figure 3B, *p* value is 0.000). Then, 50% cell cytotoxic concentrations (CC50) of the venom of *C. andromeda* and *C. mosaicus* was 2 and 10 mg/mL, respectively, for A549 and was 17 and 18 mg/mL, respectively, for HFF cells (Figure 3C,D).

### 2.5. Western Blot Assay Findings

**Figure 4:**
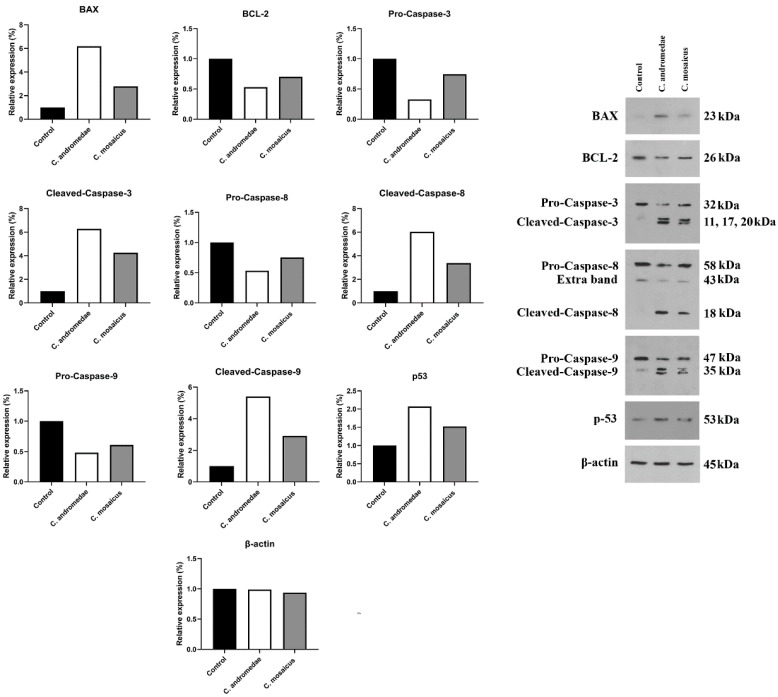
Western blot analysis of the effects of 2 and 10 mg/mL concentration of the venom of *Cassiopea andromeda* and *Catostylus mosaicus*, respectively, on the apoptotic and pre-apoptotic factors in A549 cells.

The A549 cell line was treated with a 2 mg/mL concentration of the venom of *C. andromeda*. The expression of Bcl-2-associated X protein (BAX), Tumor protein P53 (P53), cleaved-caspase-3, -8, and -9 increased in A549 cell line after treatment (Figure 4). However, the expression of B-cell lymphoma 2 (BCL-2), pro-caspase-3, -8, and -9 decreased in A549 cell line after treatment (Figure 4).

Furthermore, the A549 cell line treated with 10 mg/mL concentration of the venom of *C. mosaicus*. The level of the expression of some factors, including BAX, P53, cleaved-caspase-3, -8, and -9 was raised and a reduction in the expression of BCL-2, pro-caspase-3, -8, and -9 was observed after treatment (Figure 4). The expression of B-actin in A549 cell line was considered as the control of Western blot analysis in this study.

### 2.6. Cnidocytes and Cnidosomes: Histology and Histomorphology

**Figure 5:**
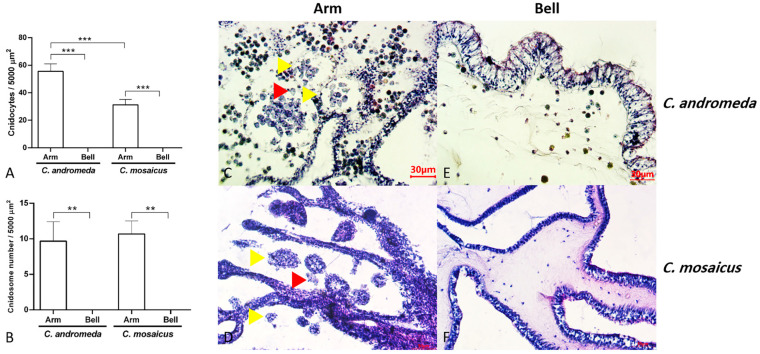
Populations of cnidocytes and cnidosomes in the arms and bells of *Cassiopea andromeda* and *Catostylus mosaicus.* (**A**,**B**) comparisons of the number of cnidocytes and cnidosomes. Lines show significant differences. ** *p* < 0.05, *** *p* < 0.01. **C–F**: Tissue section of arms and bells to show cnidocytes (red arrow head) and cnidosomes (yellow arrow head). Hematoxylin and eosin (H&E) staining.

Evaluation of tissue slides from two jellyfish, *C. andromeda* and *C. mosaicus*, showed the presence of cnidocytes with their sting-like structure and ball-shaped cnidosomes in the arm of jellyfish (Figure 5). Cnidocytes are specialized cells in the structure of the arm of *C. andromeda* and *C. mosaicus* and as we remarked before, they contain a sting-like structure. They exist in a vast number in the arms of both *C. andromeda* and *C. mosaicus* (Figure 5A). On the other side, the population of cnidocytes was seen in the structure of the bells of *C. andromeda* and *C. mosaicus* was lower than the arms of mentioned jellyfishes (Figure 5A).

**Figure 6:**
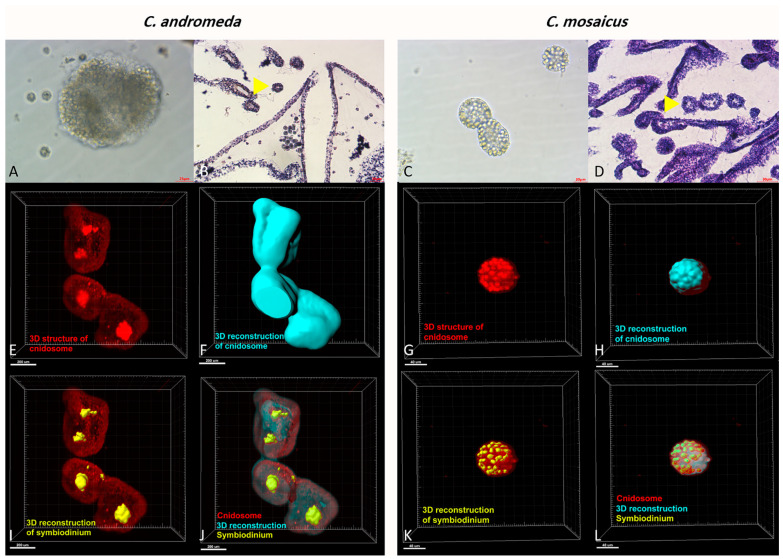
Cnidosomes in the mucus and bells of *Cassiopea andromeda* and *Catostylus mosaicus*. (**A**) An isolated cnidosome from mucus of *C. andromeda* under stereomicroscope and (**B**) tissue section of arms to show cnidosomes of *C. andromeda* (yellow arrow head). H&E staining. (**C**) An isolated cnidosome from mucus of *C. mosaicus* under stereomicroscope, (**D**) Tissue section of arms to show cnidosomes of *C. mosaicus* (yellow arrow head). H&E staining. Electroluminescence imaging (**E**–**L**), light sheet fluorescence microscopy (LSFM) imaging of cnidosomes and 3D reconstruction of them released in the mucus of the *C. andromeda* and *C. mosaicus* and their related symbiodiniums in the structure of cnidosomes.

Additionally, the observation of cnidosomes under light microscopes and light sheet microscope revealed that they contain lots of cnidocytes in their spherical structure (Figure 6). Moreover, these cnidosomes have flagella-like structures, which exist on the exterior part of their surface. Besides, symbiodiniums locate in the structure of both mentioned jellyfishes and utilize cnidosomes as their habitat (Figure 6).

In addition, the observation of the mucus of *C. andromeda* and *C. mosaicus* under a light microscope demonstrated that the irritation of both mentioned jellyfishes results in the secretion of a large amount of mucus by them. This mucus, secreted by both *C. andromeda* and *C. mosaicus,* contained a large number of cnidosomes, which had motility and floated along the mucus (Video S1).

## 3. Discussion

### 3.1. Venom of C. andromeda and Venom of C. mosaicus Have Anti-Cancer Effects on A549 Cells

Jellyfish are poisonous marine animals with some special structures used for manufacturing, storing and injecting toxins in order to capture preys and defend against strikers. The venom of jellyfish has undergone different types of surveys and only a small part of their vast biological functions has been clarified until today. Other than interesting effects like hemolytic, neurotoxic and myotoxic activity, mentioned venoms have anti-inflammatory, anti-microbial, anti-oxidant and anti-tumor effects.

The MTT assay in the current survey proves that both venoms of *C. andromeda* and *C. mosaicus* have a suppressive effect on the proliferation of A549 cells in a dose-dependent manner. While the anticancer effects of the crude venom of *C. andromeda* have been confirmed against other types of cancers like breast cancer [14].

Based on previous studies, the protein concentration of the *C. andromeda* crude venom using the Brad-ford method was found to be 17.5 mg/mL [24]. The protein concentration of the *C. mosaicus* crude venom using the Coomassie protein assay kit, was found to be 1.7 mg/gram of tissue [25]. The SDS-PAGE analysis of the *C. andromeda* crude venom with 12% acrylamide gel, showed 11 major and fewer minor protein bands, with approximate molecular weights of 12 to 225 kDa [24]. The SDS-PAGE analysis of the *C. mosaicus* crude venom with 10 or 12.5% acrylamide gels, showed four prominent and fewer minor protein bands, with approximate molecular weights of 80 to 106 kDa [25]. Not only the tiny organic-soluble compounds of the *C. andromeda* crude venom are tentatively identified by GC-MS analysis, but also the venom hydrophilic proteins constituents of remarked venom have been shown. In better words, the role of organic compounds in jellyfish venom, including proteins and enzymes and their role in stopping cell division or inducing apoptosis in cancer cells, has been shown [14]; tiny organic-soluble compounds tentatively identified by GC-MS analysis can also play an anti-cancer role [26,27].

This research has shown that the CC50 for the venom of *C. andromeda* and *C. mosaicus* were 2 and 10 mg/mL for A549. These two concentrations had the most suppressive effect on A549 and the least inhibitory influence on HFF cell lines. Earlier surveys proved that both mentioned venoms have lethal activity and some life-threatening influences on rat and mouse models in a dose-dependent manner. For instance, in a survey on the effect of the venom of *C. andromeda* on the hematological parameters in a rat model, it was demonstrated that the venom of *C. andromeda* has diverse effects, including necrotizing and hemorrhaging effects on derm. Moreover, rectal and eye bleeding and some post injection behavioral effects like confusion, aggression and violence were seen [28]. However, some studies have presented the haemolytic, edematogenic and haemorrhagic activities of the venom of *C. mosaicus* in a mouse model [29]. Despite remarked facts about the lethality of both mentioned venoms, former investigations demonstrated that, when the venoms of cnidarians are diluted and their concentration reaches lower than their lethal level, they show remarkable anti-tumoral effects with lesser fatal side effects [30].

Another finding in the present study is the proliferative effect of either the venom of *C. andromeda* on HFF cells in the concentration of 0.05 mg/mL or the venom of *C. mosaicus* on A549 and HFF cells in the concentrations of 0.05 and 1.5 mg/mL and mentioned findings were statistically significant. Consistent with our findings, the increase in cell numbers has been observed in lower concentrations than the anti-proliferative levels of cnidarian venoms on cancer cell lines are demonstrated [15,31]. For example, the venom of *Nemopilema nomurai* displayed proliferative effects on human breast cancer (MCF-7), epithelial human breast cancer (MDA-MB231) and human colon cancer (HT29) cell lines [15]. Furthermore, the venom of five sea anemones, including *Heteractis crispa*, *Heteractis magnifica*, *Heteractis malu*, *Cryptodendrum adhaesivum* and *Entacmaea quadricolor,* showed a non-significant increase in cell number on A549, human epidermoid carcinoma (A431) and human breast cancer (T47D) cell lines in dose and time-dependent manners [31]. To the best of our knowledge, the accurate mechanism(s) of this finding has not been revealed yet.

According to the present research, the venom extract of *C. andromeda* and *C. mosaicus* had five and six compounds with anti-cancer biological effects, respectively. Ethaneperoxoic acid, 1-cyano-1-[2-(2-phenyl-1,3-dioxolan-2-yl)ethyl]pentyl ester and citrinin are two components in the venom of *C. andromeda* and *C. mosaicus,* respectively, which have been proved to have anticancer activity against tumor cell lines [32,33]. Furthermore, citrinin activates some important factors involved in apoptosis, including p21-activated protein kinase 2 (PAK2) dependent pathways, caspase-3 and -9, c-Jun N-terminal kinase (JNK) and it raises reactive oxygen species (ROS) [33]. Caspase-3 is famous as an executioner caspase in apoptosis. It plays a crucial role in coordinating the disruption of cellular structures, such as DNA fragmentation or the degradation of cytoskeletal proteins [34]. Caspase-9 is a cysteine-aspartic protease with the initiation of intrinsic apoptosis, regulating physiological cell death and pathological tissue degeneration as its major functions in cell cycle arrest [35]. Ultimately, by means of all the mentioned activities, citrinin triggers apoptosis in human cancer cells.

Interestingly, there are four mutual anticancer compounds between the venom of *C. andromeda* and *C. mosaicus*. Some of them have been displayed to have anti-tumor functions independently, but some of them exist in the structure of diverse extracts like essential oil, crude extracts, etc. Dibutyl phthalate is one of the mutual components in both venoms of the mentioned jellyfishes with an interesting biological activity against different sorts of cell lines like brain, liver, lung, cervix, and colon [36]. The other three components are 9,12-octadecadienoic acid, methyl ester, heptadecane,2,6,10,14-tetramethyl- and batilol that all of them demonstrated cytotoxic influences against various cancer cell lines (Tables S1 and S2). *Melastomastrum capitatum* is a sort of traditional herbal medicine to cure diverse kinds of maladies, especially ovarian cancer. Octadecadienoic acid, methyl ester exists in the methanol fractions of the leaf of *Melastomastrum capitatum* (Vahl) Fern. Moreover, octadecadienoic acid, methyl ester can suppress human lung cancer cell lines [37]. Linear saturated diterpene (heptadecane,2,6,10,14-Tetramethyl) had high affinity to cannabinoid receptor type 1 (CB1). Induction of CB1 receptor expression leads to anticancer effects [38]. Anti-proliferative and pro-apoptotic role of CB1 receptor in human colon cancer cell lines has been shown [39]. The biological activity of heptadecane,2,6,10,14-tetramethyl- has been examined in recent studies. This compound is one of the main components of the essential oil of *Grewia Lasiocarpa* E. Mey. Ex Harv. (Malvaceae), an indigenous South African plant, and this essential oil has a cytotoxic influence against Hela cells [40]. The methanolic extract of *Chlorella vulgaris*, which contains heptadecane,2,6,10,14-tetramethyl- as one of its subsets, can target the breast cancer cell line (MCF-7) [41]. Batilol is the other mutual component between the venom of *C. andromeda* and *C. mosaicus* with anticancer activities and it is also available in the methanol solvent extraction of sea urchin [42].

The other compound, which can be found in venoms of *C. andromeda* and *C. mosaicus,* is nonadecane. This remarkable component, existing in the essential oil of *Anethum graveolens*, is an annual medicinal plant belonging to the family Umbelliferae that has an essential oil that has strong antioxidant activity [43]. According to the information collected from this study, the crude venom of *C. andromeda* and *C. mosaicus* has the ability to act against A549 cells and also possesses the potential to be used in developing anticancer agents.

### 3.2. The Venom of C. andromeda and C. mosaicus Induced Apoptosis in A549 Cells

**Figure 7:**
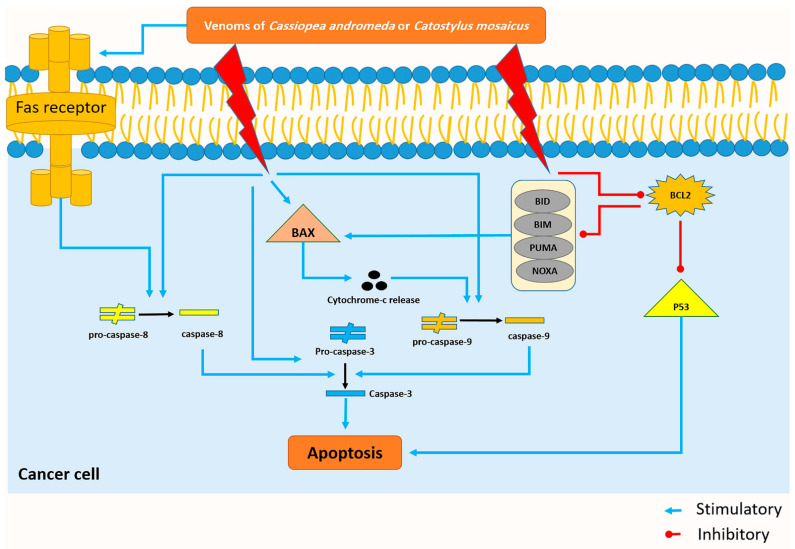
The venom of *Cassiopea andromeda* and *Catostylus mosaicus* induces the apoptosis pathway in the A549 cell lines. Blue and red lines represent venom-induced stimulus and upregulating reaction, respectively.

The cell viability of A549 cells decreased by both venoms of *C. andromeda* and *C. mosaicus*. One of the essential parts of the cell cycle is apoptosis, which is defined as a caspase-mediated programmed cell death and has the responsibility for controlling the proliferation of cells. It stops abnormal cells that contain irreversibly damaged DNA so that different lacks and disruptions exist in the process of apoptosis in cancer cells [44]. In line with the present results, previous studies of both jellyfish have revealed that the crude venom of *C. andromeda* had some interesting biological activities on human breast adenocarcinoma cells. Increasing mitochondrial ROS production, inducing cytochrome c release, MMP collapse, mitochondrial swelling and activating caspase-3 are some of these biological effects by which the mentioned venom could induce apoptosis and increase cell death in the mentioned cancer cells [18]. In the present survey, BAX, P53, cleaved-caspase-3, -8, and -9 increased and BCL-2, pro-caspase-3, -8, and -9 decreased in A549 cell line after treatment with both venoms of *C. andromeda* and *C. mosaicus* (Figure 7).

The BCL-2 family plays a crucial role in the intrinsic apoptosis pathway. The BCL-2 family contains two class of proteins: Pro-apoptotic proteins like Bax, Bcl-Xs, Bcl-2 homologous antagonist killer (Bak) and anti-apoptotic proteins such as BCL-2, Bcl-W, Bcl-XL [45]. The balance between the mentioned pro-apoptotic and anti-apoptotic proteins cause a situation in which other essential functions in normal cells are done without any problem. However, this balance is disrupted by the increase in the BCL-2 level in cancer cells, and subsequently, the inhibition of apoptosis is the main result of this disturbance [46].

Bax, which was upregulated by the toxin of both mentioned jellyfishes, has the capability to release cytochrome-c, and subsequently, activation of some apoptotic factors like caspase-9; this enzyme can also activate caspase-3 and cell apoptosis will occur at the end of this cascade (Figure 7) [47].

Activation of caspase-8, which plays a crucial role in the extrinsic pathway of apoptosis, is conducted by the activation of the Fas-receptor and the activation of caspase-8 results in the activation of caspase-3 and the happening of apoptosis (Figure 7) [48]. Interestingly, the present study displays that citrinin, which exists in the venom of *C. mosaicus*, has a high affinity to mentioned receptor (Table 2). BCL-2 can inhibit the intrinsic pathway of apoptosis by suppressing the effects of some BH3-only BCL-2 proteins, including BIM (BCL-2-like11), BID (BH3 interacting domain death agonist), PUMA (p53 upregulated modulator of apoptosis), NOXA (phorbol-12-myristate13-acetate-induced protein 1) whose activation eventually ends to turning the BAX on and occurring of apoptosis (Figure 7) [49]. Moreover, it can disrupt the activity of P53 and create an anti-apoptotic condition in cells (Figure 7) [50]. In better words, in normal cells, P53 suppresses nuclear factor kappa-light-chain-enhancer of activated B cells (NF-κB) and induces apoptosis to keep the balance between cell proliferation and cell death but in cancer cells, BCL-2 downregulates the activity of P53 and demolishes mentioned balance and leads to uncontrolled proliferation [51].

However, studies have proved that inactivation of the caspase family is an important feature of cancer cells. Caspase-3 is one of the most important members of the caspase family that needs to be cleaved and activated by granzyme B or caspase-10. The activation of caspase-3 results in the degradation of intracellular structural proteins and functional proteins and induces cell death [52]. Caspase-8 and -9 are other members of the caspase family and their major role in the process of the induction of apoptosis in various cancer cells in in vitro and in vivo either solely or in accompanying with caspase-3 has been demonstrated by prior researches [53]. Notably, previous studies have introduced BCL-2, caspase-3 and p53 as the most important genes involved in apoptosis [54]. As diverse studies have displayed previously, P53 and its vast functions like DNA repair, metabolic adaptation, cell cycle arrest, cell senescence and cell death can lead cancer cells to apoptosis and ultimately death [55].

The results of the present survey demonstrated that treatment of the A549 cell lines via both venoms of *C. andromeda* and *C. mosaicus* activated pro-apoptotic pathways and suppressed anti-apoptotic pathways. It was shown that the mentioned toxins utilize p53 and BCL-2 genes, which caused DNA fragmentation in order to trigger cell apoptosis and subsequently, induced cell death [56].

### 3.3. Molecular Docking and MD Simulation Findings Showed Two Molecules with Anti-Cancer Effect

A complex with lower binding affinity (kcal/mol) is more stable, so a ligand–protein conformation with the lowest biding affinity is favorable. The binding affinity values lower than −4 were assigned to good interactive relationships between ligand and target. In this regard, nearly all identified compounds showed good affinity to the targets. However, citrinin and ethaneperoxoic acid, 1-cyano-1-[2-(2-phenyl-1,3-dioxolan-2-yl)ethyl]pentyl ester showed better affinity to targets. It can be attributed to their structure and their intermolecular interactions with targets, including van der Waals interactions, hydrophobic interactions and hydrogen bonds. In better words, among all ligands investigated by means of molecular docking, two ligands (citrinin and ethaneperoxoic acid, 1-cyano-1-[2-(2-phenyl-1,3-dioxolan-2-yl)ethyl]pentyl ester showed better affinity to targets) showed the highest affinity to target receptors.

As displayed in Figure 2, the binding of ligands to receptors is under the control of several intermolecular interactions like van der Waals interactions, hydrophobic interactions and hydrogen bonds. Among all of these ligands, citrinin, which is a class of mycotoxins found in diverse natural sources like fruit, grains, and biological fluids with a wide range of biological effects including nephrotoxic, hepatotoxic, immunosuppression and anticancer effects [33]. As we noted in Table 2, citrinin showed the best affinity to 13 death receptors in A549 cell lines, including caspase-3 and caspase-9. However, its affinity for binding to prostaglandin D2 (PGD2) is more than other targets. Interestingly, stimulation of PGD2 results in an increase in the rate of apoptosis in lung cancer cells due to activation of the caspases pathway and synthesis of ROS (reactive oxygen species) [57].

**Figure 8:**
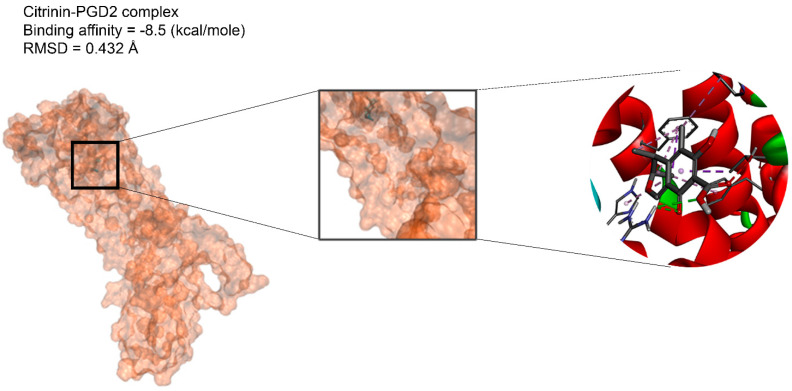
The intermolecular interactions between citrinin and PGD2.

In the next step, we performed molecular dynamic (MD) simulation in order to validate our docking findings due to some weak points of Molecular docking method including the unreliability for predicting binding energies [58]. As Molecular docking displayed that citrinin has the best affinity to 17 receptors involve in apoptosis in A549 cells (Table 2), MD simulation was conducted to confirm this finding (Table 3 and Figure 2). Between all the mentioned receptors, citrinin showed the most affinity to PGD2 (Table 2). However, the result from MD simulation was in accordance with the molecular docking findings. In better words, the RMSDs obtained from the MD simulation confirmed the results of the molecular docking (Table 2 and Table 3). However, according to the results of the MD simulation, the L structure of the complex of citrinin-PGD2 is the most stable form of the mentioned complex with RMSD of 0.432 Å (Figure 2 and Figure 8). Since citrinin is a component with diverse functions, its features have been studied by different studies [59]. In other words, citrinin has been investigated in in silico condition before in order to examine its cytotoxic activity [60].

However, ethaneperoxoic acid, 1-cyano-1-[2-(2-phenyl-1,3-dioxolan-2-yl)ethyl]pentyl ester displayed high affinity to six death receptors, including caspase-8, cannabinoid receptor type 2 (CB2) and endothelial protein C receptor (EPCR) and its highest affinity was related to the CB2 receptor and EPCR. Prior surveys have demonstrated that activation of CB2 receptor leads to the inhibition of tumor cells as it can suppress some crucial cancer cell activities, such as activation, proliferation and motility [61]. Omega-6 fatty acid ester (9,12-octadecadienoic acid, methyl ester) and stearyl monoglyceride (batilol) had high affinity to the EPCR. On the contrary, EPCR is famous because of its anti-apoptotic and carcinogenesis roles in different cancer cells to the extent that it has the capability to promote the invasiveness, metastasis, migration and proliferative strength of tumoral cells, activate anti-apoptotic pathways and facilitate angiogenesis of cancer in the body of the patient [62].

Accordingly, ethaneperoxoic acid, 1-cyano-1-[2-(2-phenyl-1,3-dioxolan-2-yl)ethyl]pentyl ester and citrinin found in the venom of *C. andromeda* and *C. mosaicus,* respectively, can be presented as two fascinating anti-cancer components of the toxin of both remarked jellyfishes. This result is in accordance with those that have been reported in the inhibitory properties of citrinin against the growth and proliferation of different cancer cells such as sarcoma and those who introduce 1-cyano-1-[2-(2-phenyl-1,3-dioxolan-2-yl)ethyl]pentyl ester as a potential antitumor compound that can be used against diverse tumors [63,64].

### 3.4. C. andromeda and C. mosaicus Venoms Can Be Isolated from Cnidocytes and Cnidosomes

In line with prior research, the tissue section of the arm of *C. andromeda* and *C. mosaicus* in the present study demonstrated that both jellyfishes have a large number of cnidocytes with spike-like structures and are very sensitive to mechanical and physical irritation [65]. However, in this article, we demonstrated that the main concentration of cnidocytes is in the arm of both jellyfishes. In better words, the concentration of cnidocytes in the arm of both jellyfish is more than its concentration in the bell (Figure 5). This fact is in opposition to some of the previous surveys in which the distribution of cnidocytes has been reported in all parts of the structure of cnidarians. The concentration of cnidocytes is varied in various sorts of cnidarians. Some cnidarians have more cnidocytes in the structure of their bell and some of them have cnidocytes all over their structure [66].

In addition, the tissue slides of *C. andromeda* and *C. mosaicus* demonstrated that both jellyfish have cnidosomes in their structure and their cnidosomes consist of cnidocytes. The same finding has been reported for *C. andromeda* [67]. Additionally, the observation of cnidosomes in the mucus released by jellyfishes present in this study showed that they have flagella on their surface and they move along the mucus which is released by both jellyfishes and they are released from the arms of jellyfish under the influence of environmental stimuli. Furthermore, light sheet fluorescence microscopy (LSFM) helped us to find out the location of symbiodinium in the structure of cnidosomes of both jellyfishes. As we displayed for symbiodinium in Figure 6, they locate in the center and in the peripheral surface of cnidosomes in *C. andromeda* and *C. mosaicus*, respectively.

## 4. Materials and Methods

### 4.1. Jellyfish Collection and Adaptation

The current study was conducted based on relevant guidelines and regulations of animal studies and all experimental protocols were approved by the ethical committee of Bushehr University of Medical Sciences with the ethical code of IR.BPUMS.REC.1399.186. *C. andromeda* and *C. mosaicus* were, respectively, gathered from the Nayband mangrove forest and the Delvar intertidal zone in the Persian Gulf, during the summer of 2020. After the collection of five adult samples of each, they were transferred to the Marine Comparative and Experimental Medicine of the Bushehr University of Medical Sciences. The identities of *C. andromeda* and *C. mosaicus* were verified by Dr. Amir Vazirizadeh from the Department of Marine Biotechnology, the Persian Gulf Research and Studies Center, the Persian Gulf University, Bushehr, Iran [24].

### 4.2. Histological Distribution of Cnidocytes and Cnidosomes in Jellyfish Body

In order to examine the histological distribution of cnidocytes and cnidosomes in the body of jellyfish, the methods developed for Staurozoa were chosen for histological procedures [68]. In details, the tissue samples were fixed into artificial seawater containing 4% formaldehyde and it was substituted with a fresh solution after one week. Then, the slides of tissues of both jellyfish underwent the tissue processing step. The process was initiated by dehydration in 70%, 80%, 96% and 100% ethanol series, respectively, and after that they were cleared by xylene in two separate steps. Afterwards, they were immersed in paraffin twice for 75 and 45 min. After that, the sectioning was performed transversely with the thickness of 7.0–10.0 μm using a Leica RM 2025 rotatory microtome (Leica Microsystems, Wetzlar, Germany). Sections of the tissue of both jellyfish were transferred to the laboratory for hematoxylin and eosin (H&E) staining. In order to conduct the process of H&E staining, the slides of both jellyfish tissue were placed into xylene two times and after that, they were put into 100%, 96% and 76% ethanol twice for each concentration of ethanol, respectively. Afterwards, the slides of both tissues were put in hematoxylin and then they were placed in an acid fuchsin solution. Next, they were transferred into eosin dye and after that they were rinsed with distilled water. After mentioned processes, slides of both tissues of jellyfish were placed in 76%, 96%, 100% ethanol and xylene twice for each step, respectively. Between each stage, the slides were transferred into distilled water for enhancing the contrast between the structures. Finally, the prepared slides were watched and pictured via a light microscope (OPTIKA, Modena, Italy) and a microscope camera (OMAX, A35180U3, Gyeonggi-do, Korea). The slides were kept in the collection of the Persian Gulf Marine Biotechnology Research Center, the Persian Gulf Biomedical Sciences Research Institute, Bushehr University of Medical Sciences and are available for further analysis.

### 4.3. Isolation of Cnidocytes and Cnidosomes in Jellyfish Body

Cnidocytes were isolated from the *C. andromeda* and *C. mosaicus,* as explained by Choudhary et al. [69], with slight modifications. Briefly, *C. andromeda* and *C. mosaicus* underwent an excision process and their oral arms were excised. After excision, oral arms were transferred to two separate Erlenmeyer flasks, which contained 300 mL of sterile seawater. After labeling, two flasks were placed on plastic dishes, which were full of pieces of crushed ice and they were daily shaken for 45 min. This process was repeated for 4 days. After the shaking process, the content of each flask was collected and transferred into a falcon tube. Falcon tubes were centrifuged at 4000× *g* at 4 °C for 10 min and settled material was resuspended in fresh seawater. The mentioned process was repeated once daily for two more days. Afterwards, the sediments were collected and underwent centrifugation at 200× *g* at 4 °C for 5 min, and then the seawater was removed and each falcon tube was filled with 10× phosphate-buffered saline (PBS). After that, the undischarged cnidocytes were collected and some of them were lyophilized and stored at −70 °C until used. Some of them were fixed directly in a 4% formaldehyde solution with seawater for further analysis.

Cnidosomes isolation was performed through the method explained by Ames et al. [67]. In brief, artificial seawater was filtered and *C. andromeda* and *C. mosaicus* were placed into two separate glass dishes containing filtered artificial seawater. Translocation of jellyfish into dishes made them to secrete mucus within 2 to 5 min. After releasing mucus, cnidosomes of *C. andromeda* and *C. mosaicus* were separately collected via Pasteur pipette and using a stereo microscope (Cobra Micro Zoom, MZ1000, Micros, Sankt Veit an der Glan, Austria). Isolated cnidosomes were pictured via a light microscope (OPTIKA, Modena, Italy) and a microscope camera (OMAX, A35180U3, Gyeonggi-do, Korea).

In order to fix cnidosomes for light sheet microscopy imaging, we performed the method of Ames et al. [67] with a little modification. First, both jellyfishes were transferred into a container including 300 mL of sea water and irritated gently for releasing their mucus into the sea water. In the next step, their mucus was separated from seawater by pipettor and transferred into two separate petri dishes. After collecting cnidosomes from the mucus of both jellyfishes with a Pasteur pipette under a light microscope, we Fixed cnidosomes in 4% paraformaldehyde in PBS for 1 h at 4 °C. After that, cnidosomes underwent three times washing in PBS with 1% in order to remove fixative from the cnidosomes. In the next step, cnidosomes were embedded into a 1.5% low melting point agarose. Ultimately, fixed cnidosomes were transferred into two syringes for light sheet microscopy imaging.

### 4.4. Light Sheet Imaging of Jellyfish Cnidosomes

An open spim platform was employed to acquire all the light-sheet fluorescent microscope (LSFM) [70] images using 405 and 473 nm lasers were utilized in the manner of the light sources for fluorescence and bright field imaging, respectively. An Olympus 10×/0.3 water dipping type was chosen as the illumination objective. A tube lens and a 0.5× camera lens accompanied with a 20×/0.5 water dipping objective (Olympus equipment) were used as a detection arm. In order to set the position of the samples inside the field of view, the bright field modality was used. To pass the fluorescence signals at the range of 417–477 nm, a DAPI filter was utilized. The thickness of the sheet was set to 20 μm and 200 images were gathered at a step size of 2 μm for each z-stack. A three-time repeat was performed for each image so that a voxel size in all dimensions could be 0.7 μm. The amount of lateral and axial resolution of the system was 1 μm and 10 μm, respectively. A LabVIEW program was employed for data acquisition. For preparing samples, they were fixed in 1% w/v agarose gel in a sample chamber full of pure water. Clusters with more thickness floated in a borosilicate capillary, which was attached to a syringe while inside the capillary. Moreover, the sample chamber was filled with a sorbitol refractive index matching solution. Finally, Imaris software was used for the purpose of rendering the three-dimensional (3D) models.

### 4.5. Imaris Reconstruction of Light Sheet Images of Jellyfish Cnidosomes

First, serial images of the LFSM microscope were imported into ImageJ software (ImageJ, National Institutes of Health, Bethesda, MD, USA). After that, the remarked serial images were combined as a TIFF series image using “Images to Stack” tool in “Image” panel. In the next step, the TIFF series image was saved in TIFF format. Then, Imaris software (V 7.4.2, ImarisX64, Bitplane AG) was elected to conduct 3D reconstruction of LFSM images of cnidosomes. Firstly, serial TIFF images of cnidosomes of *C. mosaicus* and *C. andromeda* were reconstructed using “Surfaces” algorithm. Then, the “Surfaces” algorithm was used for the second time with different colors in order to reconstruct other structures. The blue and yellow, “Surfaces”, algorithm was used to reconstruct the whole tissue of cnidosomes and symbiodinium structures, respectively.

### 4.6. Venom Isolation from Cnidocytes of Jellyfish

The venom of *C. andromeda* and *C. mosaicus* were achieved from freeze-dried cnidocytes utilizing the technique explained by Seymour et al. with a slight modification [71]. Briefly, 50 mg of lyophilized cnidocytes powder was weighted for each jellyfish and it was dissolved in 1 mL of cold PBS (pH 7.4, 4 °C). The mixtures were shaken at 3000 rpm for 30 s, separately. This shaking process was repeated 10 times with ice cooling periods between each repetition. After the end of the whole shaking process, two micro tubes were centrifuged at 15,000× *g*, 4 °C for 30 min. After centrifugation, the supernatant of each mixture with the concentration of 50 mg/mL was achieved and used as venom for the next steps.

### 4.7. A549 Cell Culture and Treatment with Venoms of C. andromeda and C. mosaicus

A549 and HFF cells (PerciaVista Biotech Co., Shiraz, Iran) were cultured in a culture media which included Dulbecco’s Modified Eagle Medium (DMEM, Gibco, Life Technologies Co., Carlsbad, NM, USA), 10% fetal bovine serum (FBS, Kiazist, Tehran, Iran) and 1% penicillin-streptomycin (Pen-Strep, Gibco, Life Technologies Co., Carlsbad, NM, USA). The cell lines were seeded in T-75 flasks at 37 °C in a humidified atmosphere with 5% CO_2_. When both A549 and HFF cell lines reached approximately 90% of confluency, the cells were passaged and collected from the bottom of each flask in order to perform the seeding process in 96 dish-well plates.

One day after the preformation of the seeding process, preparation of the venoms of *C. andromeda* and *C. mosaicus* were conducted, as explained before. Different concentrations of the venoms were separately prepared by dilution of the venom with DMEM, 10% FBS and 1% Pen-Strep via the first concentration (100 mg/mL) including 0.05, 0.15, 1.5, 5, 10 and 50 mg/mL. Then, cells were treated with remarked concentrations for both venoms. In the next step, cells were incubated for three days at 37 °C in a humidified atmosphere with 5% CO_2_.

After that, for cell vitality assessment, the media were removed and 100 µL of MTT (3-(4,5-dimethylthiazol-2-yl)-2,5-diphenyltetrazolium bromide) assay kit (Sigma-Aldrich Co., Darmstadt, Germany) with the concentration of 5 mg/mL was added to each well and incubated for 4 h until the intracellular purple formazan crystals appeared. Then, DMSO was added to each well and incubated at 37 °C and 5% CO_2_ for 20 min. The cell absorbance of each plate was read at a wavelength of 573 nm using an ELISA plate reader machine (BioTek, Winooski, VT, USA). This step was repeated three times.

In the next step, cell viability was obtained through the formula below [72]:

$$\%\mathrm{Viable}\;\mathrm{cells}=\frac{Mean\;\mathrm{Optical}\;\mathrm{Density}\;\left(\mathrm{OD}\right)\;of\;sample}{Mean\;\mathrm{Optical}\;\mathrm{Density}\;\left(\mathrm{OD}\right)\;of\;blank}\times 100$$

Then, 50% cell cytotoxic concentrations (CC50) of *C. andromeda* and *C. mosaicus* were calculated via nonlinear regression of “log (inhibitor) vs. normalized response” in Graphpad prism (v7.0a, GraphPad Software, Inc., San Diego, CA, USA).

### 4.8. GC-MS Analysis of Venoms of C. andromeda and C. mosaicus

Both lyophilized crude venoms underwent the extraction process by means of methanol: Chloroform: n-hexane (2:2:1 *v*/*v*), and after that, they were introduced to the gas chromatography-mass spectroscopy (7890B Agilent GC-MS, Santa Clara, CA, USA). Mass spectra were taken at filament emission of 0.5 Ma, a scanning interval of 0.5 sec and fragments from m/z 50 to 500 Da and ionizing energy 70 Ev. An HP-5MS UI capillary column (30 m × 0.25 mm ID × 0.25 µm) was employed for the GC separation. Helium gas was used as the carrier gas at a constant flow rate of 0.8 mL/min, injection volume of 1ulit and split ratio of 30:1. The temperatures of the transfer line, injection port and ion-source were 250 °C, 240 °C and 270 °C. The temperature of the oven was set at 80 °C for 3 min and after that, it started to increase by 5 °C/min until reaching 250 °C and was held for 10 min. The total time of GC running was 38 min and 22 min, for *C. andromeda* and *C. mosaicus*, respectively. In order to define separated compounds, the National Institute of Standards and Technology (NIST MS database. 2014) library was used. Eventually, by comparing the average pick area of each composition with total areas, the relative amount (%) of components was measured.

### 4.9. Western Blot Analysis of Apoptosis Pathways in A549 Cells after Exposure to Venoms of C. andromeda and C. mosaicus

Western blot analysis was done based on standard procedures with slight modifications [73]. Based on the findings of the cell MTT assay, the most effective concentrations of the venoms were determined and used for Western blot analysis in the A549 cell line. In details, after 72 h cell exposure to extracts, cells were lysed by RIPA buffer including 500 µL Tris–HCl (pH = 8.0), 0.003 gr ethylenediaminetetraacetic acid (EDTA), 0.08 gr NaCl, 0.025 gr sodium deoxycholate, 0.01 gr sodium dodecyl sulfate (SDS), 1 tablet protease inhibitor cocktail and 10 µL triton (NP40). The lysates were centrifuged at 12,000 ×g for 10 min at 4 °C, and the supernatant containing the protein was extracted and stored at −20 °C. After that, the protein concentration in the supernatant was measured by a Bradford protein assay. Proteins were then transferred to a microporous polyvinylidene difluoride membrane (Millipore, France). Membranes were incubated in 5% bovine serum albumin (BSA, Sigma, USA) blocking buffer for 1 h at room temperature. After blocking, the membranes were incubated with the corresponding primary antibodies, separately overnight at 4 °C. Immunoblotting was performed with rabbit anti-β-actin (sc-47778, 1:300), anti- Bax (sc-7480, 1:300), anti-Bcl2 (sc-492, 1:300), anti-caspase-3 (sc-136219, 1:300), -3, and -7 antibodies (1:200) (Cell Signaling Technology, Danvers, CO, USA). After the previous step, the paper was washed three times with a TBST buffer for 15 min each time.

The paper was then quenched with anti-rabbit secondary antibody at a concentration (1:1000) for all primary antibodies for 1 h and 15 min at room temperature. At the end of this step, the paper was washed three times with a TBST buffer for 15 min each time. After the final washing of the previous step, the excess water of PVDF paper was placed on the cellophane and the chemiluminescence solution was poured with a sampler on the desired band areas. The paper was wrapped in cellophane and inserted into the film cassette.

### 4.10. Molecular Interactions and Docking Studies of A549 Apoptotic Pathways and Effective Molecules of Jellyfish’ Venoms

After finding the most known death receptors that are involved in the process of apoptosis in A549 cells and their role in the remarked process (Table S3), 17 death receptors were chosen to undergo a molecular docking process (Table 1). However, the GC-MS determined components in the venom of *C. andromeda* and *C. mosaicus.* Then, based on previous studies, compounds in both mentioned venoms with the capability to affect cancer cells were selected to be utilized in the molecular docking process (Table 2 and Table S1). Autodock Vina 1.1.2, a molecular docking tool, was used to seek the precise binding site of ligands on protein. The 3D structures of seven components and 17 key targets (caspase-3, caspase-7, caspase-8, caspase-9, CB1, CB2, DR4, EPCR, Fas receptor, insulin like growth factor 1 receptor (IGF1R), metabotropic glutamate receptors (mGluRs), peroxisome proliferator-activated receptor-γ (PPAR-γ), transforming growth factor beta receptor 2 (TGFBR2), toll-like receptor 4 (TLR4), toll-like receptor 9 (TLR9), tumor necrosis factor receptor 1 (TNFR1), and PGD2 with protein data bank (PDB) code of 4jje, 1f1j, 4ps1, 4rhw, 6kpg, 6kpf, 5cir, 1lqv, 3tje, 3lw0, 6bsz, 7m8w, 1fm6, 4g8a, 3wpc, and 1ft4) were downloaded from the PUBCHEM and PDB databases, respectively.

In order to prepare for further docking analysis, chimera was used to prepare targets, non-standard residuals were omitted and hydrogen atoms were added at first. Then, nonpolar hydrogens and ione pairs were merged and a Gasteiger partial charge was given to each atom. In order to generate grid boxes, their size and position were selected by using the Computed Atlas of Surface Topography of proteins (CASTp 3.0). After performing docking, 10 conformations were obtained for each target and ligand. All docking conformations were ranked according to the score (binding affinity) and the best one was selected based on the lower negative energy and RMSD ≤ 2 Å.

### 4.11. MD Simulation Studies for Effective Molecules of Jellyfish’ Venoms

Molecular dynamics simulations were performed for 100 ns for the compound with the highest affinity using the NAMD2 and VMD (version 1.9.3) to validate the docking process.

## 5. Conclusions

*C. andromeda* and *C. mosaicus* are equipped with specialized structures that give the ability to create the different biological effects on the live environment surrounding. Present survey displays that both venoms of *C. andromeda* and *C. mosaicus* have the ability to inhibit cancer cells in a dose-dependent manner. The ideal dose of the venom of *C. andromeda* and *C. mosaicus* for suppressing A549 cells is 2 and 10 mg/mL, respectively. Moreover, our findings display some components in the structure of the venom of both remarked jellyfish, which have remarkable biological anticancer activity against human pulmonary adenocarcinoma, one of the most challenging malignancies all over the world. This research showed that, in addition to macromolecule organic compounds, such as proteins, peptides and enzymes in the venoms that have been confirmed in the previous studies, there are tiny organic compounds in the venom of *C. andromeda* and *C. mosaicus* that can have anti-cancer effects on pulmonary adenocarcinoma through apoptotic and anti-proliferative pathways. The isolation of these molecules in the future may lead to the development of new therapeutic agents for pulmonary adenocarcinoma.

## Figures and Tables

**Table 1:** GC-MS-detected compounds with an anticancer property in the venom of *Cassiopea andromeda* (*CA*) and *Catostylus mosaicus* (*CM*).

Jellyfish	No.	Compounds	Formula	MW	RT	Abundance Ratio (%) *	
						*CA*	*CM*
*Cassiopea andromeda*/*Catostylus mosaicus*	1	Dibutyl phthalate	C_16_H_22_O_4_	278.34	18.41	3.153	0.746
	2	9,12-Octadecadienoic acid, methyl ester	C_19_H_34_O_2_	294.5	21.86	4.852	5.552
	3	Heptadecane,2,6,10,14-Tetramethyl-	C_21_H_44_	296.6	24.4	0.775	5.221
	4	Batilol	C_21_H_44_O_3_	344.6	30.14	0.506	1.397
*Cassiopea andromeda*	5	Ethaneperoxoic acid, 1-cyano-1-[2-(2-phenyl-1,3-dioxolan-2-yl)ethyl]pentyl ester	C_19_H_25_NO_5_	347.4	15.94	1.098	NA
*Catostylus mosaicus*	6	Citrinin	C_13_H_14_O_5_	250.25	17.33	NA	4.402
	7	Eicosane	C_20_H_42_	282.5	19.29	NA	1.269

* Abundance ratio, the average peak area of each compound per total peaks area.

**Table 2:** The values of binding affinity (Kcal/mol) of ligands to the receptors that are involved in the process of apoptosis in human pulmonary adenocarcinoma cells (A549).

Ligands	Receptors																
	Caspase-3	Caspase-7	Caspase-9	CB1	CB2	DR4	EPCR	Fas Receptor	IGF1R	mGluRs	PPAR-γ	TGFBR2	TLR4	TLR9	TNFR1	PGD2	Caspase-8
9,12-Octadecadienoic acid, methyl ester	−5.2	−5.3	−4.6	−6.7	−7.3	−3.5	−7.6	−3.8	−5.6	−4.9	−5.3	−4.9	−4.4	−5.5	−3.9	−7	−5.5
Batilol	−5.4	−5.6	−3.7	−6.3	−4.3	−3.2	−7.3	−3.9	−4.6	−5.2	−5.6	−4.2	−4.4	−4.8	−3.9	−6.6	−5.6
Citrinin	−6.8	−6.4	−5.3	−7.8	−7.8	−6	−8.1	−6.5	−6.4	−5.9	−7.6	−5.6	−6.6	−6.8	−5.8	−8.5	−6.1
Dibutyl phthalate	−5.5	−5.5	−4.3	−7.6	−5.1	−4.4	−7.7	−5.1	−5.8	−5.5	−6.2	−4.8	−5.1	−5.2	−4.6	−7.8	−6
Eicosane	−4.6	−4.4	−3.5	−6.8	−4.4	−3	−7.4	−3.2	−4.6	−3.7	−5.2	−4.4	−4.3	−4.3	−3.3	−6.6	−4.8
Ethaneperoxoic acid, 1-cyano-1-[2-(2-phenyl-1,3-dioxolan-2-yl)ethyl]pentyl ester	−6.7	−6.6	−4.4	−6.9	−8.1	−5.6	−8.1	−5.4	−6.4	−6.2	−7.5	−5.3	−5.8	−6.5	−5.4	−8.4	−6.8
Heptadecane,2,6,10,14-Tetramethyl-	−4.5	−5.1	−3.7	−7.3	−4.9	−3.4	−8	−4	−5.6	−4.3	−6.1	−4.7	−4.6	−5.7	−4.1	−7.1	−5.5

**Table 3:** RMSDs of citrinin targets obtained from molecular dynamics.

Receptors	Avg	SD	Min	Max
caspase-3	0.530	0.077	0.422	0.638
caspase-7	0.621	0.102	0.453	0.768
caspase-8	0.633	0.058	0.551	0.718
caspase-9	0.821	0.081	0.702	0.938
CB1	0.464	0.062	0.367	0.549
CB2	0.461	0.070	0.356	0.558
DR4	0.685	0.061	0.604	0.769
EPCR	0.451	0.062	0.364	0.535
Fas Receptor	0.601	0.104	0.464	0.744
IGF1R	0.628	0.062	0.524	0.738
mGluRs	0.739	0.070	0.636	0.838
PGD2	0.432	0.060	0.312	0.538
PPAR-γ	0.482	0.062	0.388	0.572
TGFBR2	0.795	0.079	0.679	0.914
TLR4	0.555	0.070	0.454	0.654
TLR9	0.540	0.60	0.454	0.647
TNFR1	0.729	0.90	0.614	0.838

## Data Availability

Data are contained within the article or Supplementary Materials.

## Supplementary Materials

The following supporting information can be downloaded at: https://www.mdpi.com/article/10.3390/md21030168/s1: Figure S1: GC-MS chromatogram of the venom of *Cassiopea andromeda* and *Catostylus mosaicus*; Table S1: GC-MS compounds in the venom of *Cassiopea andromeda*; Table S2: GC-MS compounds in the venom of *Catostylus mosaicus*; Table S3: Receptors involve in the apoptosis of cells of human pulmonary adenocarcinoma cells (A59); Western blot original gels [32,33,36,43,74,75,76,77,78,79,80,81,82,83,84,85,86,87,88,89,90,91,92,93,94,95,96,97,98,99,100,101,102,103,104,105,106,107,108,109,110,111]. Video S1: Cnidosomes of *Cassiopea andromeda* and *Catostylus mosaicus* and their motility through the mucus secreted by the jellyfish. Video S2: 3D reconstructed images of cnidosomes of *Cassiopea andromeda* and *Catostylus mosaicus* after imaging by LSFM.
