# Supplementary material for: Chemical Compositions and Experimental and Computational Modeling of the Anticancer Effects of Cnidocyte Venoms of Jellyfish Cassiopea andromeda and Catostylus mosaicus on Human Adenocarcinoma A549 Cells
Source: Mar Drugs. 2023 Mar 7;21(3):168. doi: 10.3390/md21030168 (PMC10057638; doi:10.3390/md21030168)
Supplement: Supplementary file 1 [file marinedrugs-21-00168-s001.zip › marinedrugs-1982838-supplementary-12.2.pdf]

**Chemical compositions and experimental and computational modeling of anticancer effects of cnidocyte venoms of jellyfish *Cassiopea andromeda* and *Catostylus mosaicus* on human adenocarcinoma A549 cells**

Afshin Zare[1], Alireza Afshar[2], Arezoo Khoradmehr[3], Neda Baghban[3], Gholamhossein Mohebbi[3], Alireza Barmak[4], Adel Daneshi[3], Afshar Bargahi[3], Iraj Nabipour[3], Sahar Almasi-turk[5,*], Alireza Arandian[6], Mohammad Ismail Zibaii[6], Hamid Latifi[6,7], Amin Tamadon[2,*]

1- Bushehr University of Medical Sciences, Bushehr, Iran
2- PerciaVista R&D Co. Shiraz, Iran
3- The Persian Gulf Marine Biotechnology Research Center, The Persian Gulf Biomedical Sciences Research Institute, Bushehr University of Medical Sciences, Bushehr, Iran
4- Food Lab, Bushehr University of Medical Sciences, Bushehr, Iran
5- Department of Anatomical Sciences, School of Medicine, Bushehr University of Medical Sciences, Bushehr, Iran
6- Laser and Plasma Research Institute, Shahid Beheshti University, Tehran, Iran
7- Department of Physics, Shahid Beheshti University, Tehran, Iran

*Correspondences:
Amin Tamadon, DVM, PhD; The PerciaVista Biotechnology Company, Shiraz, Iran; Postal Code: 7514633196; Tel/fax: +98-21-2842-6122; Email: amintamaddon@yahoo.com
Sahar Almasi-turk, PhD; Department of Anatomical Sciences, Bushehr University of Medical Sciences, Bushehr, Iran; Postal Code: 7518759577; Tel./fax: +98-77-3332-0657 Email: s.almasi@bpums.ac.ir

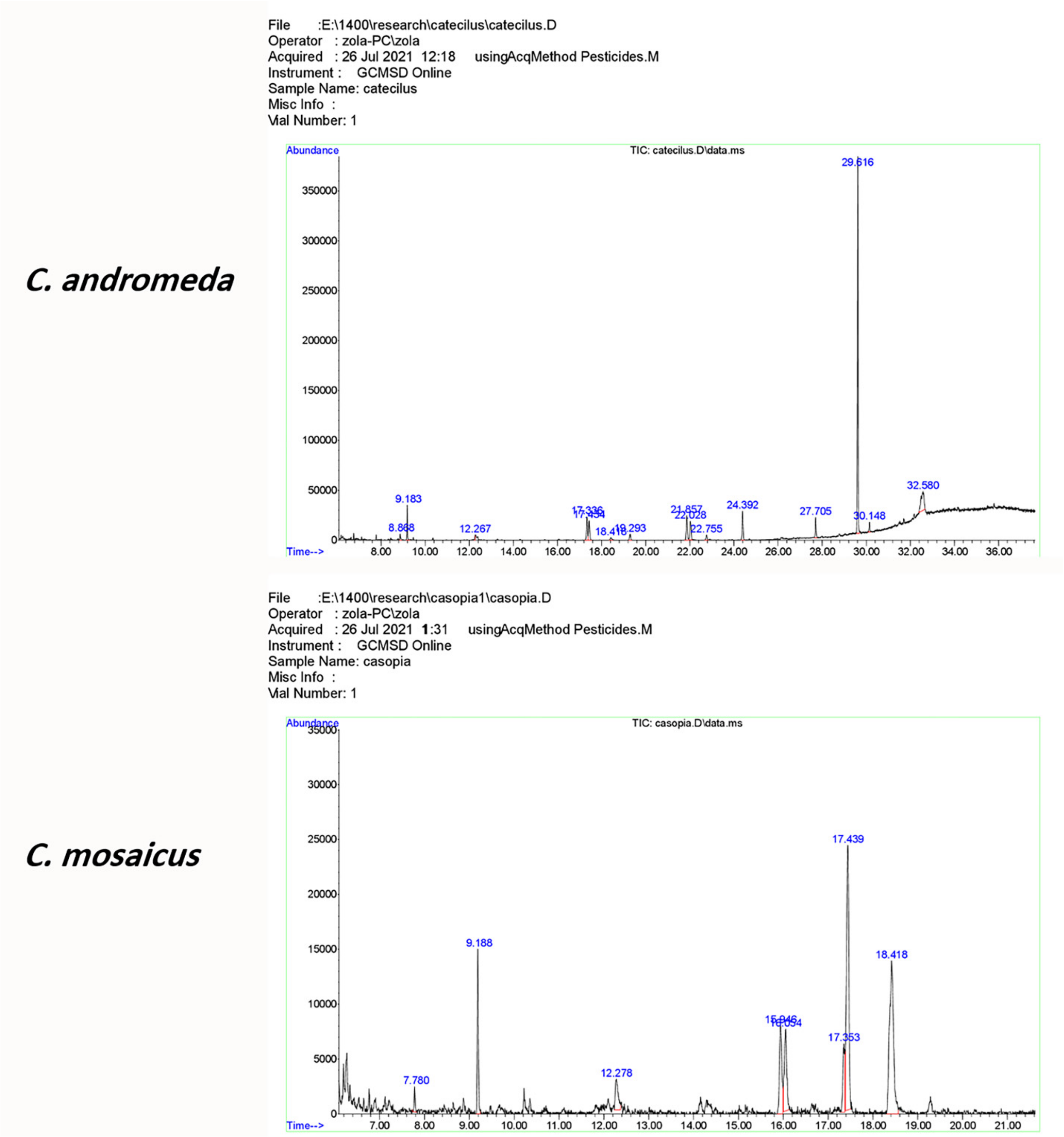


**Figure S1.**
GC-MS chromatogram of the venom of *Cassiopea andromeda* and Catostylus *mosaicus*

**Table S1.**
GC-MS-detected compounds in the venom of *Cassiopea andromeda*

| Compounds | Formula | Effects | MW | RT | Abundance ratio (%) | Cancer/Cell line | References |
|---|---|---|---|---|---|---|---|
| Ethaneperoxoic acid, 1-cyano-1-[2-(2-phenyl-1,3-dioxolan-2-yl)ethyl]pentyl ester | C19H25NO5 | Anticancer<br>Antimicrobial<br>Anti-inflammatory | 347.4 | 15.94 | 1.098 | MCF-7 | [1-3] |
| Dibutyl phthalate | C16H22O4 | Anticancer<br>Antimicrobial<br>Induce apoptosis<br>Induce inflammation<br>Induce oxidative stress | 278.34 | 18.41 | 3.153 | Brain<br>Breast<br>Lung<br>Prostate<br>Bladder | [4-6] |
| 9,12-Octadecadienoic acid, methyl ester | C19H34O2 | Anticancer<br>Antioxidant<br>Antimicrobial<br>Anti-inflammatory | 294.5 | 21.86 | 4.852 | B16<br>Eca-109<br>BGC823<br>HeLa<br>NCTC1469 | [7-10] |
| 6-Octadecenoic acid, methyl ester, (Z)- | C19H36O2 | Antioxidant<br>Antimicrobial<br>Antiviral | 296.5 | 22.03 | 3.575 | NA | [11-13] |
| Heptadecane,2,6,10,14-Tetramethyl- | C21H44 | Anticancer<br>Antioxidant | 296.6 | 24.4 | 0.775 | LnCap<br>22RV1<br>MDA-MB-231<br>MCF7 | [14] |
| 1-Hexadecyne | C16H30 | Antimicrobial | 222.41 | 26.13 | 0.451 | NA | [15] |

| | | | | | | | |
|---|---|---|---|---|---|---|---|
| Nonadecane | C19H40 | Antioxidant Anti-inflammatory | 268.5 | 27.7 | 1.176 | NA | [16] |
| Batilol | C21H44O3 | Anticancer Anti-inflammatory | 344.6 | 30.14 | 0.506 | Leukemia | [17,18] |
| 2-Bromononane | C9H19Br | NA | 207.15 | 7.78 | 0.123 | NA | NA |
| Tetradecanoic acid, 12-methyl-, methyl ester, (S)- | C16H32O2 | NA | 256.42 | 22.77 | 0.880 | NA | NA |
| Androst-11-en-17-one, 3-formyloxy-, (3a',5a')- | C20H28O3 | NA | 316.4 | 31.14 | 4.701 | NA | NA |
| Pseduosarsasapogenin-5,20-dien | C27H42O3 | NA | 414.6 | 32.6 | 29.621 | NA | NA |
| 1,3-Dioxolane-2-heptanenitrile, alpha-methyl-e-oxo-2-phenyl- | C17H21NO3 | NA | 287.35 | 16.05 | 0.998 | NA | NA |
| Aspidofractinine-3-methanol, (2a',3a',5a')- | C22H28N2O2 | NA | 352.5 | 29.62 | 42.856 | NA | NA |
| 1,4,5,6-Tetrahydrocyclopentapyrazole-3-carboxylic acid, (1-adamantan-1-ylethylidene)hydrazide | C19H26N4O | NA | 326.4 | 9.18 | 0.927 | NA | NA |
| 3β-hydroxyallobetulane | C30H50O2 | NA | 442.7 | 17.43 | 3.165 | NA | NA |
| 3-oxo-18-nor-ent-ros-4-ene-15β,16-acetonide | C22H34O3 | NA | 346.5 | 17.35 | 0.687 | NA | NA |
| Cholestan-22(26)-isoepoxy | C27H42O1 | NA | 414.6 | 12.27 | 0.457 | NA | NA |

**Table S2.**
GC-MS-detected compounds in the venom of *Catostylus mosaicus*

| Compounds | Formula | Effects | MW | RT | Abundance ratio (%) | Cancer/Cancer cell line | References |
|---|---|---|---|---|---|---|---|
| Citrinin | $C_{13}H_{14}O_5$ | Anticancer<br>Antioxidant<br>Antimicrobial<br>Anti-inflammatory<br>Induce apoptosis<br>Induce oxidative stress<br>Antiviral | 250.25 | 17.33 | 4.402 | MCF-7<br>LNCaP<br>LU-1<br>KB | [19-24] |
| Dibutyl phthalate | $C_{16}H_{22}O_4$ | Anticancer<br>Antimicrobial<br>Induce apoptosis<br>Induce inflammation<br>Induce oxidative stress | 278.34 | 18.41 | 0.746 | Brain<br>Breast<br>Lung<br>Prostate<br>Bladder | [4-6] |
| Eicosane | $C_{20}H_{42}$ | Anticancer<br>Antimicrobial<br>Anti-inflammatory<br>Antiviral | 282.5 | 19.29 | 1.269 | HeLa<br>MCF7<br>SGC-7901 | [25-29] |
| 9,12-Octadecadienoic acid, methyl ester | $C_{19}H_{34}O_2$ | Anticancer<br>Antioxidant<br>Antimicrobial | 294.5 | 21.85 | 5.552 | B16<br>Eca-109<br>BGC823 HeLa<br>NCTC1469 | [7-10] |

| | | | | | | | |
|---|---|---|---|---|---|---|---|
| 6-Octadecenoic acid, methyl ester, (Z)- | C19H36O2 | Anti-inflammatory Antioxidant Antimicrobial | 296.5 | 22.03 | 4.480 | NA | [11-13] |
| Heptadecane,2,6,10,14-Tetramethyl- | C21H44 | Anticancer Antioxidant | 296.6 | 24.4 | 5.221 | LnCap 22RV1 MDA-MB-231 MCF7 | [14] |
| Nonadecane | C19H40 | Antioxidant | 268.5 | 27.7 | 3.297 | NA | [16] |
| Batilol | C21H44O3 | Anti-inflammatory Anticancer Anti-inflammatory | 344.6 | 30.14 | 1.397 | Leukemia | [17,18] |
| Decane, 1-bromo-2-methyl- | C11H23Br | NA | 235.20 | 8.86 | 0.791 | NA | NA |
| 3β-hydroxyallobetulane | C30H50O2 | NA | 442.7 | 17.43 | 3.961 | NA | NA |
| Aspidofractinine-3-methanol, (2a',3a',5a')- | C22H28N2O2 | NA | 352.5 | 29.62 | 52.603 | NA | NA |
| Tetradecanoic acid, 12-methyl-, methyl ester, (S)- | C16H32O2 | NA | 256.42 | 22.76 | 1.145 | NA | NA |
| 1,4,5,6-Tetrahydrocyclopentapyrazole-3-carboxylic acid, (1-adamantan-1-ylethylidene)hydrazide | C19H26N4O | NA | 326.4 | 9.18 | 3.912 | NA | NA |
| Cholestan-22(26)-isoepoxy | C27H42O1 | NA | 414.6 | 12.26 | 0.609 | NA | NA |
| Pseduosarsasapogenin-5,20-dien | C27H42O3 | NA | 414.6 | 32.6 | 10.616 | NA | NA |

**Table S3.**
Receptors involve in the apoptosis of cells of human pulmonary adenocarcinoma cells (A59).

| **Receptor** | **Type of effect on the apoptosis in A549 cells** | **Ref.** |
|---|---|---|
| Fas receptor | Activation | [30] |
| TNF receptor-1(TNF-R1) | Activation | [30] |
| Death receptor 4 (DR4 or TRAIL-RI) | Activation | [31] |
| Insulin-like growth factor 1 receptor (IGF1R) | Activation | [32] |
| Peroxisome proliferator-activated receptor-$\gamma$ (PPAR-$\gamma$) | Activation | [33] |
| Caspase-8 | Activation | [34] |
| Caspase-3 | Activation | |
| Caspase-9 | Activation | |
| Caspase-7 | Activation | [35] |
| Cannabinoid receptor type 1 (CB1) | Activation | [36] |
| Cannabinoid receptor type 2 (CB2) | | |
| Toll-like receptor 4 (TLR4) | Activation | [37] |
| Toll-like receptor 9 (TLR9) | Activation | [38] |
| Blockade of Endothelial protein C receptor (EPCR) | Inhibition | [39] |
| Metabotropic glutamate receptors (mGluRs) | Activation | [40] |
| Prostaglandin D2 (PGD2) receptor | Activation | [41] |
| Transforming Growth Factor Beta Receptor 2 (TGFBR2) | Activation | [42] |

**Western blot original gels.**

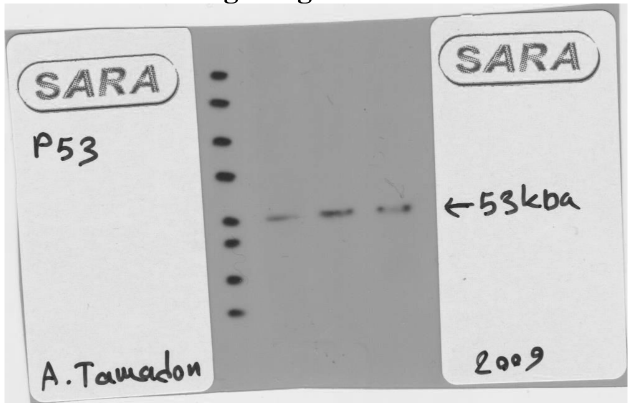


Western blot gel P53

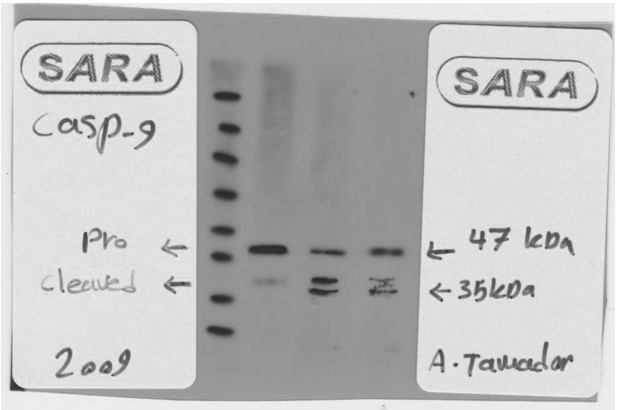


Western blot gel caspase-9

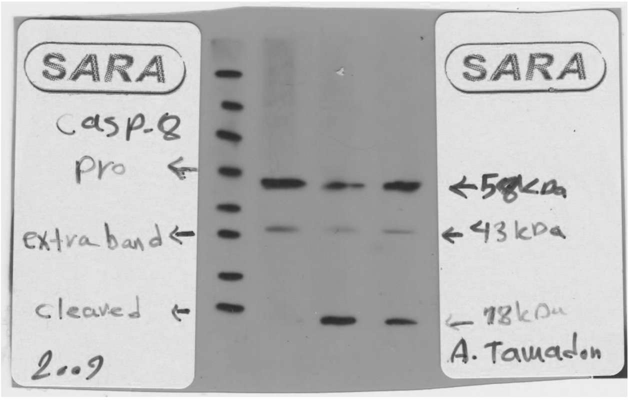


Western blot gel caspase-8

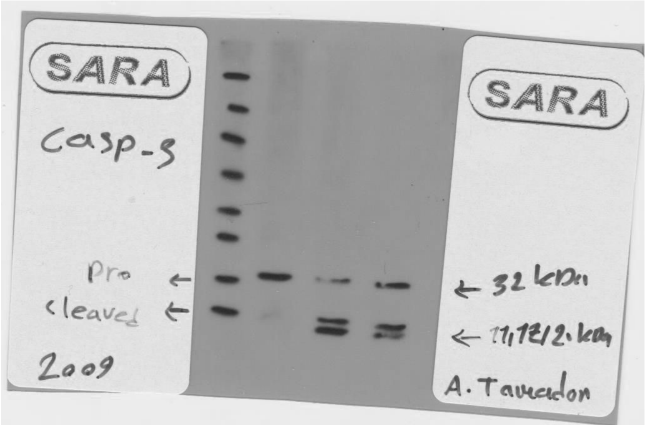


Western blot gel caspase-3

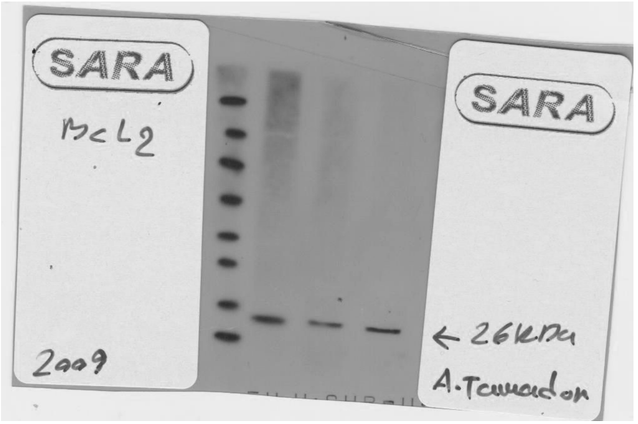


Western blot gel BCL-2

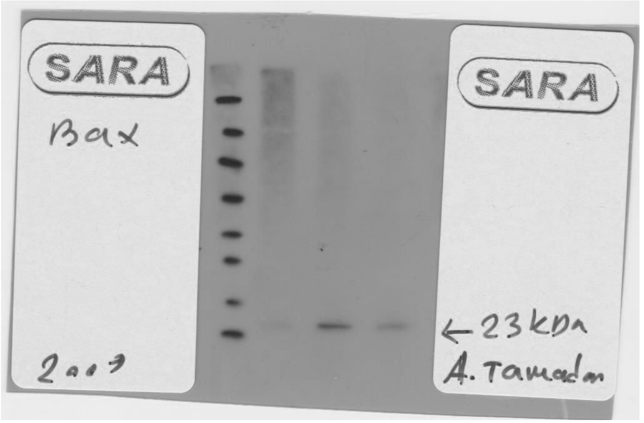


Western blot gel BAX

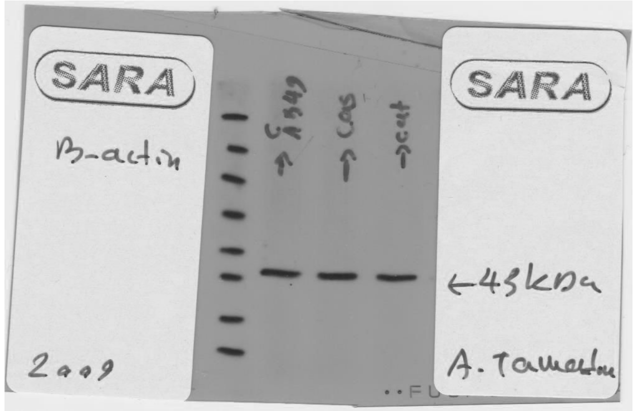


Western blot gel β-actin

References


1. Sathyaprabha, G.; Panneerselvam, A.; Kumaravel, S. Bioactive compounds identification of *Pleurotus platypus* and *Pleurotus eous* by GC-MS. *Adv. Appl. Sci. Res.* **2011**, *2*, 51-54.
2. Bekele, D.; Tekie, H.; Asfaw, Z.; Petros, B. Bioactive chemical constituents from the leaf of *Oreosyce africana* Hook. f (Cucurbitaceae) with mosquitocidal activities against adult *Anopheles arabiensis*, the principal malaria vector in Ethiopia. *J. fertil. pestic.* **2016**, *7*, doi:10.4172/2471-2728.1000159.
3. Maharajasri, V.; Devamalar, D. Characterization of antibacterial, anticancer properties and bioactive compounds of methanolic leaf extract of *Catharanthus roseus*. *Int. J. Humanit. Arts Med. Sci.* **2015**, *1*, 35-42.
4. Roy, R.N. Bioactive natural derivatives of phthalate ester. *Crit. Rev. Biotechnol.* **2020**, *40*, 913-929, doi:10.1080/07388551.2020.1789838.
5. Malik, S.K.; Ahmed, M.; Khan, F. Identification of novel anticancer terpenoids from *Prosopis juliflora* (Sw) DC (Leguminosae) pods. *Trop. J. Pharm. Res.* **2018**, *17*, 661-668, doi:10.4314/tjpr.v17i4.14.
6. Cui, Y.; Zhang, X.; Yin, K.; Qi, X.; Zhang, Y.; Zhang, J.; Li, S.; Lin, H. Dibutyl phthalate-induced oxidative stress, inflammation and apoptosis in grass carp hepatocytes and the therapeutic use of taxifolin. *Sci. Total Environ.* **2021**, *764*, 10, doi:10.1016/j.scitotenv.2020.142880.
7. Yang, Y.; Fu, C.; Zhou, F.; Luo, X.; Li, J.; Zhao, J.; He, J.; Li, X.; Li, J. Chemical composition, antioxidant and antitumor activities of sub-fractions of wild and cultivated *Pleurotus ferulae* ethanol extracts. *PeerJ* **2018**, *6*, e6097, doi:10.7717/peerj.6097.
8. Sutanto, H.; Ainny, L.; Susanto, B.H.; Nasikin, M. Reaction product of pyrogallol with methyl linoleate and its antioxidant potential for biodiesel. In Proceedings of the IOP Conference Series: Materials Science and Engineering, 2018; p. 012019.
9. Batalha, M.M.C.; Goulart, H.F.; Santana, A.E.G.; Barbosa, L.A.O.; Nascimento, T.G.; da Silva, M.K.H.; Dornelas, C.B.; Grillo, L.A.M. Chemical composition and antimicrobial activity of cuticular and internal lipids of the insect *Rhynchophorus palmarum*. *Arch. Insect. Biochem. Physiol.* **2020**, *105*, e21723, doi:10.1002/arch.21723.
10. El-anssary, A.A.; Raoof, G.F.A.; Saleh, D.O.; El-Masry, H.M. Bioactivities, physicochemical parameters and GC/MS profiling of the fixed oil of *Cucumis melo* L seeds: A focus on anti-inflammatory, immunomodulatory, and antimicrobial activities. *J. HerbMed Pharmacol.* **2021**, *10*, 476-485, doi:10.34172/jhp.2021.55.
11. Marie Elvire, N.; Hortense, G.; Emmanuel, N.; Rose, N.; Francine, M. Evaluation of the in vitro antibacterial activity of *Azadirachta indica* used for the treat-ment of alveolitis. *Int. J. Biopharm. Sci.* **2020**, *2*, 119, doi:10.31021/ijbs.20203119.
12. Dadwal, V.; Agrawal, H.; Sonkhla, K.; Joshi, R.; Gupta, M. Characterization of phenolics, amino acids, fatty acids and antioxidant activity in pulp and seeds of high altitude *Himalayan crab* apple fruits (Malus baccata). *J. Food Sci. Technol.* **2018**, *55*, 2160-2169, doi:10.1007/s13197-018-3133-y.

13. Khiralla, A.; Spina, R.; Varbanov, M.; Philippot, S.; Lemiere, P.; Slezack-Deschaumes, S.; Andre, P.; Mohamed, I.; Yagi, S.M.; Laurain-Mattar, D. Evaluation of antiviral, antibacterial and antiproliferative activities of the endophytic fungus *Curvularia papendorfii*, and isolation of a new polyhydroxyacid. *Microorganisms* **2020**, *8*, 1353, doi:10.3390/microorganisms8091353.
14. El Fakir, L.; Bouothmany, K.; Alotaibi, A.; Bourhia, M.; Ullah, R.; Zahoor, S.; El Mzibri, M.; Gmouh, S.; Alaoui, T.; Zaid, A. Antioxidant and understanding the anticancer properties in human prostate and breast cancer cell lines of chemically characterized methanol extract from *Berberis hispanica* Boiss. & Reut. *Appl. Sci.* **2021**, *11*, 3510, doi:10.3390/app11083510.
15. Adenola, O.; Adeleke, S. Determination of bioactive compounds and antimicrobial capabilities of purified *Nymphaea lotus* Linn. (Nymphaeaceae) extract to multidrug resistant enteric bacteria. *J. Altern. Complement. Med.* **2021**, *13*, 39-53, doi:10.9734/JOCAMR/2021/v13i230222.
16. Kazemi, M. Phenolic profile, antioxidant capacity and anti-inflammatory activity of *Anethum graveolens L.* essential oil. *Nat. Prod. Res.* **2015**, *29*, 551-553, doi:10.1080/14786419.2014.951934.
17. Burford, R.G.; Gowdey, C.W. Anti-inflammatory activity of alkoxyglycerols in rats. *Arch. Int. Pharmacodyn. Ther.* **1968**, *173*, 56-70.
18. Ge, G.F.; Yu, C.H.; Yu, B.; Shen, Z.H.; Zhang, D.L.; Wu, Q.F. Antitumor effects and chemical compositions of *Eupolyphaga sinensis* walker ethanol extract. *J. Ethnopharmacol.* **2012**, *141*, 178-182, doi:10.1016/j.jep.2012.02.016.
19. de Oliveira Filho, J.W.G.; Islam, M.T.; Ali, E.S.; Uddin, S.J.; Santos, J.V.O.; de Alencar, M.; Junior, A.L.G.; Paz, M.; de Brito, M.; JMC, E.S.; et al. A comprehensive review on biological properties of citrinin. *Food Chem. Toxicol.* **2017**, *110*, 130-141, doi:10.1016/j.fct.2017.10.002.
20. Kumar, R.; Dwivedi, P.D.; Dhawan, A.; Das, M.; Ansari, K.M. Citrinin-generated reactive oxygen species cause cell cycle arrest leading to apoptosis via the intrinsic mitochondrial pathway in mouse skin. *Toxicol. Sci.* **2011**, *122*, 557-566, doi:10.1093/toxsci/kfr143.
21. Heider, E.M.; Harper, J.K.; Grant, D.M.; Hoffman, A.; Dugan, F.; Tomer, D.P.; O'Neill, K.L. Exploring unusual antioxidant activity in a benzoic acid derivative: a proposed mechanism for citrinin. *Tetrahedron* **2006**, *62*, 1199-1208, doi:10.1016/j.tet.2005.10.066
22. Sharath Babu, G.R.; Anand, T.; Ilaiyaraja, N.; Khanum, F.; Gopalan, N. Pelargonidin modulates Keap1/Nrf2 pathway gene expression and ameliorates citrinin-induced oxidative stress in HepG2 cells. *Front. Pharmacol.* **2017**, *8*, 868, doi:10.3389/fphar.2017.00868.
23. de Sousa Oliveira, K.; Queiroz, P.R.M.; Fensterseifer, I.C.M.; Migliolo, L.; Oliveira, A.L.; Franco, O.L. Purified cititin in combination with vancomycin inhibits VRE in vitro and in vivo. *Microbiology* **2017**, *163*, 1525-1531, doi:10.1099/mic.0.000547.

24. Shams ul Hassan, S.; Ishaq, M.; Zhang, W.-d.; Jin, H.-Z. An overview of the mechanisms of marine fungi-derived anti-inflammatory and anti-tumor agents and their novel role in drug targeting. *Curr. Pharm. Des.* **2021**, *27*, 2605-2614, doi:10.2174/1381612826666200728142244.
25. Vats, S.; Gupta, T. Evaluation of bioactive compounds and antioxidant potential of hydroethanolic extract of *Moringa oleifera* Lam. from Rajasthan, India. *Physiol. Mol. Biol. Plants* **2017**, *23*, 239-248, doi:10.1007/s12298-016-0407-6.
26. Saleh, E.I.M.M.; Bhattacharyya, P.; Van Staden, J. Chemical composition and cytotoxic activity of the essential oil and oleoresins of in vitro micropropagated *Ansellia africana* Lindl: A vulnerable medicinal orchid of Africa. *Molecules* **2021**, *26*, 4556, doi:10.3390/molecules26154556.
27. Elmosallamy, A.; Ibraheem, E.; Sarhan, M.; Hussein, S.A.A. Chemical and biological activities of *Deverra triradiata* Hochst. ex. Boiss. Aerial parts from St. Catherine, southern Sinai, Egypt. *Egypt. J. Chem.* **2021**, *64*, 1387-1394, doi:10.21608/EJCHEM.2020.52846.3092.
28. Okechukwu, P. Evaluation of anti-inflammatory, analgesic, antipyretic effect of eicosane, pentadecane, octacosane, and heneicosane. *Asian J. Pharm. Clin. Res.* **2020**, *13*, 29-35, doi:10.22159/ajpcr.2020.v13i4.36196
29. Chathuranga, K.; Weerawardhana, A.; Dodantenna, N.; Ranathunga, L.; Cho, W.K.; Ma, J.Y.; Lee, J.S. Inhibitory effect of *Sargassum fusiforme* and its components on replication of respiratory syncytial virus in vitro and in vivo. *Viruses* **2021**, *13*, 548, doi:10.3390/v13040548.
30. Zhao, C.; Gao, W.; Chen, T. Synergistic induction of apoptosis in A549 cells by dihydroartemisinin and gemcitabine. *Apoptosis* **2014**, *19*, 668-681, doi:10.1007/s10495-013-0953-0.
31. Lu, J.; Chen, J.; Kang, Y.; Wu, J.; Shi, H.; Fu, Y.; Jiao, L.; Dong, C.; Li, X.; Jin, Y. Jinfukang induces cellular apoptosis through activation of Fas and DR4 in A549 cells. *Oncol. Lett.* **2018**, *16*, 4343-4352, doi:10.3892/ol.2018.9149.
32. Dong, A.; Kong, M.; Ma, Z.; Qian, J.; Cheng, H.; Xu, X. Knockdown of insulin-like growth factor 1 receptor enhances chemosensitivity to cisplatin in human lung adenocarcinoma A549 cells. *Acta Biochim. Biophys. Sin.* **2008**, *40*, 497-504, doi:10.1111/j.1745-7270.2008.00429.x.
33. Mao, J.T.; Nie, W.-X.; Tsu, I.; Jin, Y.-S.; Rao, J.Y.; Lu, Q.-Y.; Zhang, Z.-F.; Go, V.L.W.; Serio, K.J. White tea extract induces apoptosis in non–small cell lung cancer cells: The role of peroxisome proliferator-activated receptor-γ and 15-lipoxygenaseswhite tea induces apoptosis in human lung cancer cells. *Cancer Prev. Res.* **2010**, *3*, 1132-1140, doi:10.1158/1940-6207.CAPR-09-0264.
34. Lin, M.; Tang, S.; Zhang, C.; Chen, H.; Huang, W.; Liu, Y.; Zhang, J. Euphorbia factor L2 induces apoptosis in A549 cells through the mitochondrial pathway. *Acta Pharm. Sin. B* **2017**, *7*, 59-64, doi:10.1016/j.apsb.2016.06.008.
35. Moghadamtousi, S.Z.; Kadir, H.A.; Paydar, M.; Rouhollahi, E.; Karimian, H. Annona muricata leaves induced apoptosis in A549 cells through mitochondrial-mediated pathway and involvement of NF-κB. *BMC Complement. Atern. Med.* **2014**, *14*, 1-13, doi:10.1186/1472-6882-14-299.

36. Preet, A.; Qamri, Z.; Nasser, M.W.; Prasad, A.; Shilo, K.; Zou, X.; Groopman, J.E.; Ganju, R.K. Cannabinoid receptors, CB1 and CB2, as novel targets for inhibition of non–small cell lung cancer growth and metastasis cannabinoid receptors in NSCLC treatment. *Cancer Prev. Res.* **2011**, *4*, 65-75, doi:10.1158/1940-6207.CAPR-10-0181.
37. Hsu, H.-Y.; Lin, T.-Y.; Lu, M.-K.; Leng, P.-J.; Tsao, S.-M.; Wu, Y.-C. Fucoidan induces Toll-like receptor 4-regulated reactive oxygen species and promotes endoplasmic reticulum stress-mediated apoptosis in lung cancer. *Sci. Rep.* **2017**, *7*, 1-13, doi:10.1038/srep44990.
38. Yuan, S.; Qiao, T.; Li, X.; Zhuang, X.; Chen, W.; Chen, X.; Zhang, Q. Toll-like receptor 9 activation by CpG oligodeoxynucleotide 7909 enhances the radiosensitivity of A549 lung cancer cells via the p53 signaling pathway. *Oncol. Lett.* **2018**, *15*, 5271-5279, doi:10.3892/ol.2018.7916.
39. Antón, I.; Molina, E.; Luis-Ravelo, D.; Zandueta, C.; Valencia, K.; Ormazabal, C.; Martínez-Canarias, S.; Perurena, N.; Pajares, M.J.; Agorreta, J. Receptor of activated protein C promotes metastasis and correlates with clinical outcome in lung adenocarcinoma. *Am. J. Respir. Crit. Care Med.* **2012**, *186*, 96-105, doi:10.1164/rccm.201110-1826OC.
40. Li, T.-J.; Huang, Y.-H.; Chen, X.; Zhou, Z.; Luo, S.-W.; Feng, D.-D.; Han, J.-Z.; Luo, Z.-Q. Metabotropic glutamate receptor 8 activation promotes the apoptosis of lung carcinoma A549 cells in vitro. *Sheng Li Xue Bao* **2015**, *67*, 513-520.
41. Wang, J.J.; Mak, O.T. Induction of apoptosis in non-small cell lung carcinoma A549 cells by PGD2 metabolite, 15d-PGJ2. *Cell Biol. Int.* **2011**, *35*, 1089-1096, doi:10.1042/CBI20110707.
42. Tarfiei, G.A.; Shadboorestan, A.; Montazeri, H.; Rahmanian, N.; Tavosi, G.; Ghahremani, M.H. GDF15 induced apoptosis and cytotoxicity in A549 cells depends on TGFBR2 expression. *Cell Biochem. Funct.* **2019**, *37*, 320-330, doi:10.1002/cbf.3391.
